# Impacts of Mg doping on the structural properties and degradation mechanisms of a Li and Mn rich layered oxide cathode for lithium-ion batteries

**DOI:** 10.1038/s41598-023-31492-0

**Published:** 2023-03-20

**Authors:** Songyoot Kaewmala, Natthapong Kamma, Sunisa Buakeaw, Wanwisa Limphirat, Jeffrey Nash, Sutham Srilomsak, Pimpa Limthongkul, Nonglak Meethong

**Affiliations:** 1 Institute of Nanomaterials Research and Innovation for Energy (IN-RIE), Khon Kaen University, Khon Kaen, 40002 Thailand; 2 Materials Science and Nanotechnology Program, Department of Physics, Faculty of Science, Khon Kaen University, Khon Kaen, 40002 Thailand; 3 National Energy Technology Center, National Science and Technology Development Agency, 111 Thailand Science Park, Phaholyothin Rd., Klong 1, Klong Luang, 12120 Pathumthani Thailand; 4 Synchrotron Light Research Institute, 111 University Avenue, Suranaree, Muang, Nakhon Ratchasima, 30000 Thailand

**Keywords:** Energy storage, Materials for energy and catalysis

## Abstract

The Li- and Mn-rich layered oxide cathode material class is a promising cathode material type for high energy density lithium-ion batteries. However, this cathode material type suffers from layer to spinel structural transition during electrochemical cycling, resulting in energy density losses during repeated cycling. Thus, improving structural stability is an essential key for developing this cathode material family. Elemental doping is a useful strategy to improve the structural properties of cathode materials. This work examines the influences of Mg doping on the structural characteristics and degradation mechanisms of a Li_1.2_Mn_0.4_Co_0.4_O_2_ cathode material. The results reveal that the prepared cathode materials are a composite, exhibiting phase separation of the Li_2_MnO_3_ and LiCoO_2_ components. Li_2_MnO_3_ and LiCoO_2_ domain sizes decreased as Mg content increased, altering the electrochemical mechanisms of the cathode materials. Moreover, Mg doping can retard phase transition, resulting in reduced structural degradation. Li_1.2_Mn_0.36_Mg_0.04_Co_0.4_O_2_ with optimal Mg doping demonstrated improved electrochemical performance. The current work provides deeper understanding about the roles of Mg doping on the structural characteristics and degradation mechanisms of Li-and Mn-rich layered oxide cathode materials, which is an insightful guideline for the future development of high energy density cathode materials for lithium-ion batteries.

## Introduction

Lithium-ion batteries have been used as energy storage devices for large-scale systems, electronic tools, and electric vehicles. This is because they exhibit higher energy density that those of other commercial battery technologies such Pd-acid, Ni–Cd, and Ni–MH batteries. The performance of lithium-ion batteries greatly depends on the electrochemical properties of their electrode materials, especially the cathode materials. Cathode materials contain Li ions in their structure. So, the electrochemical properties of cathode materials strongly affect the performance of lithium-ion batteries, including power and energy density. Currently, the utilization of Li-ion batteries has been exponentially increasing. There have been numerous efforts to explore new cathode materials with higher specific energy densities and lower costs to replace currently used commercial cathodes such as LiCoO_2_, LiFePO_4_ (LFP), and LiNi_1−x−y_Co_x_Mn_y_O_2_ (NMC) materials. Li- and Mn-rich layered oxides, xLi_2_MnO_3_·(1 − x)LiMO_2_ (M=Mn, Fe, Co, Ni, etc.), are promising cathode materials that can deliver high specific capacities of over 250 mAh/g^1–3^. However, use of this cathode material class has several problems, including low cycling stability, poor rate performance, and large voltage decay. These result from transition from a layered to a spinel-like structure during electrochemical cycling^4–7^. Previous studies reported that cation or anion substitution is an effective strategy to hinder structural transition during electrochemical cycling. Na^+^^8–10^, K^+^^11^, Al^3+^^12,13^, Ru^4+^^14,15^, Zn^2+^^16,17^, Mg^2+^^18–21^, F^−^^22–24^, and S^2−^^25^ doped Li and Mn rich layered oxide cathode materials present improved cycling stability. Wang and co-workers showed that Li_1.2_Ni_0.2_Mn_0.6_O_2_ doped by Mg in both Ni and Mn sites exhibited higher cycling stability and conductivity compared to undoped material^18^. Nayak and co-workers reported that Li_1.2_Ni_0.16_Mn_0.56_Co_0.08_O_2_ cathode materials with the proper amount of Mg doped into the Mn site revealed a retarded phase transition from a layered to a spinel structure during cycling, resulting in enhanced cycling stability^20^. Additionally, Jin and co-workers reported improved rate capability in 0.4Li_4/3_Mn_2/3_O_2_·0.6LiNi_1/3_Co_1/3_Mn_1/3_O_2_ with the appropriate amount of Mg doping in Li sites. Such doping induced expanded lithium-ion diffusion channels, facilitating Li-ion transport^21^. This indicates that Mg substitution is helpful in improving the electrochemical performance of Li- and Mn-rich layered oxide cathode materials. Although Mg doping has been used to improve the electrochemical performance of cathode materials, deeper understanding of its impacts on the structural properties and degradation behaviors during prolonged cycling is not well developed. This is essentially important for developing cathode materials to meet practical applications.

The current work aims to investigate the impacts of Mg doping on the structural characteristics and degradation mechanisms during cycling of Li_1.2_Mn_0.4_Co_0.4_O_2_ cathode materials. X-ray absorption spectroscopy (XAS) and X-ray diffraction (XRD) techniques were used to study the local atomic structure and crystal structure of the prepared Li_1.2_Mn_0.4_Co_0.4_O_2_ and Li_1.2_Mn_0.4−x_ Mg_x_Co_0.4_O_2_ (x = 0.00, 0.02, 0.04, and 0.06) cathodes, respectively. Moreover, transmission electron microscopy (TEM) was employed to examine the microstructure and morphology of the materials. The electrochemical mechanisms, electrochemical performance, and lithium-ion diffusion coefficients of the electrodes were studied using galvanostatic cycling testing and a galvanostatic intermittent titration technique (GITT). The results imply that Mg doping not only play critical roles in development of the local atomic and crystal structure as reported the previous works, but also significantly impacts the phase separation behaviors of Li_2_MnO_3_- and LiCoO_2_-like phases, altering the electrochemical mechanisms and properties of the prepared electrode materials. Furthermore, Mg doping has significant impacts on phase transformation and structural degradation mechanisms of cathode materials during cycling. Optimal Mg doping can retard spinel-like phase transition and consequently lead to improved electrochemical properties. The obtained results are an essential key to developing the electrochemical properties of Li- and Mn-rich layered oxide cathode materials.

## Results and discussion

### Morphological and microstructural characterization

**Figure 1:**
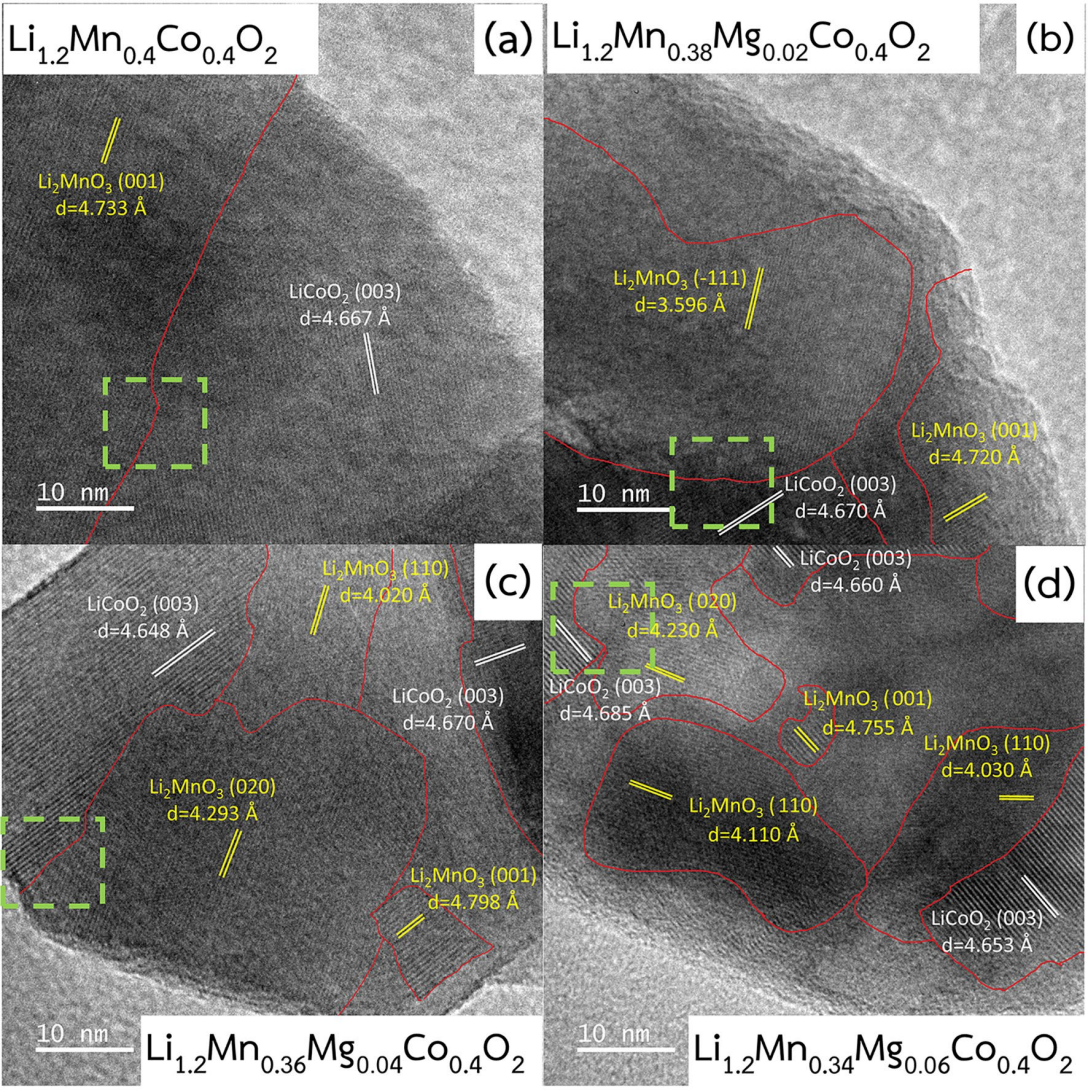
HRTEM images of the pristine Li_1.2_Mn_0.4_Co_0.4_O_2_ and Mg-doped Li_1.2_Mn_0.4_Co_0.4_O_2_materials.

Transmission electron microscopy (TEM) was employed to analyze the particle size distribution and morphology of the obtained cathode materials, as shown in Fig. S1. The prepared cathodes had broad particle size distributions of around 40 nm to 200 nm with average particle sizes of 118.00 ± 34.03 nm, 105.25 ± 28.31 nm, 107 ± 22.65 nm, and 105 ± 89 nm for Li_1.2_Mn_0.4_Co_0.4_O_2_, Li_1.2_Mn_0.38_Mg_0.02_Co_0.4_O_2_, Li_1.2_Mn_0.36_Mg_0.04_Co_0.4_O_2_, and Li_1.2_Mn_0.34_Mg_0.06_Co_0.4_O_2_, respectively. Figure 1 presents images obtained from high resolution transmission electron microscopy (HRTEM) of the synthesized materials. At least five individual particles with sizes of approximately 100 nm, which is close to the average particle size of the materials, were chosen to study by HRTEM. Li_2_MnO_3_-like and LiCoO_2_-like domains were clearly detected as monoclinic Li_2_MnO_3_ within space group $C2/m$ and rhombohedral LiCoO_2_ within space group $R\overline{3 }m$, respectively. Magnified images corresponding to the dashed green rectangles are demonstrated in Fig. S2. The LiCoO_2_ regions show a continuous atomic structure along the Li and TM layers, whereas the Li_2_MnO_3_ regions show a periodic structure associated with the presence of Li in the transition metal layers. This makes the boundaries of the regions between these two phases easily observable, which are represented by red lines that show the interfaces between the LiMO_2_ and the Li_2_MnO_3_ regions. The presence of both Li_2_MnO_3_-like and LiCoO_2_-like regions in individual particles is consistent with previous work^1,26–31^. The experimental results reflect that the prepared samples are composite materials. Our previous reports illustrated that the domain size of the Li_2_MnO_3_ phase has significant influence on the electrochemical properties of composite-based Li- and Mn-rich layered oxide cathode materials. Varying Li_2_MnO_3_ domain sizes can alter Li_2_MnO_3_ activation, largely affecting the degree of transition from a layered to a spinel structure during electrochemical cycling^30,32,33^. From our experimental results, the domain sizes of Li_2_MnO_3_-like and LiCoO_2_-like phases are strongly related to the Mg content. The domain sizes of both Li_2_MnO_3_-like and LiCoO_2_-like phases decreased as the Mg content increased, as shown in Table S1. An undoped Li_1.2_Mn_0.4_Co_0.4_O_2_ electrode had the largest Li_2_MnO_3_- and LiCoO_2_-like domain sizes, while Li_1.2_Mn_0.34_Mg_0.06_Co_0.4_O_2_, with the highest level of Mg doping, revealed the smallest Li_2_MnO_3_- and LiCoO_2_-like domain sizes. Mg doping and changes in the Li_2_MnO_3_-like domains significantly affected the electrochemical properties of the prepared materials, as seen in the electrochemical results. To confirm Mg doping, energy dispersive X-ray spectroscopy (EDS) was used to investigate elemental mapping, as shown in Fig. S3. The results reveal that the O, Mn, Co, and Mg (for Mg doped materials) species are uniformly distributed throughout the specimens. Clear particle surfaces, observed in the TEM images of Fig. S1, suggest that there were no surface coatings on the particles of the Mg-doped materials. This indicates that Mg was successfully doped into the bulk of the Mg-doped cathode materials.

### Crystal and local atomic structure characterization

**Figure 2:**
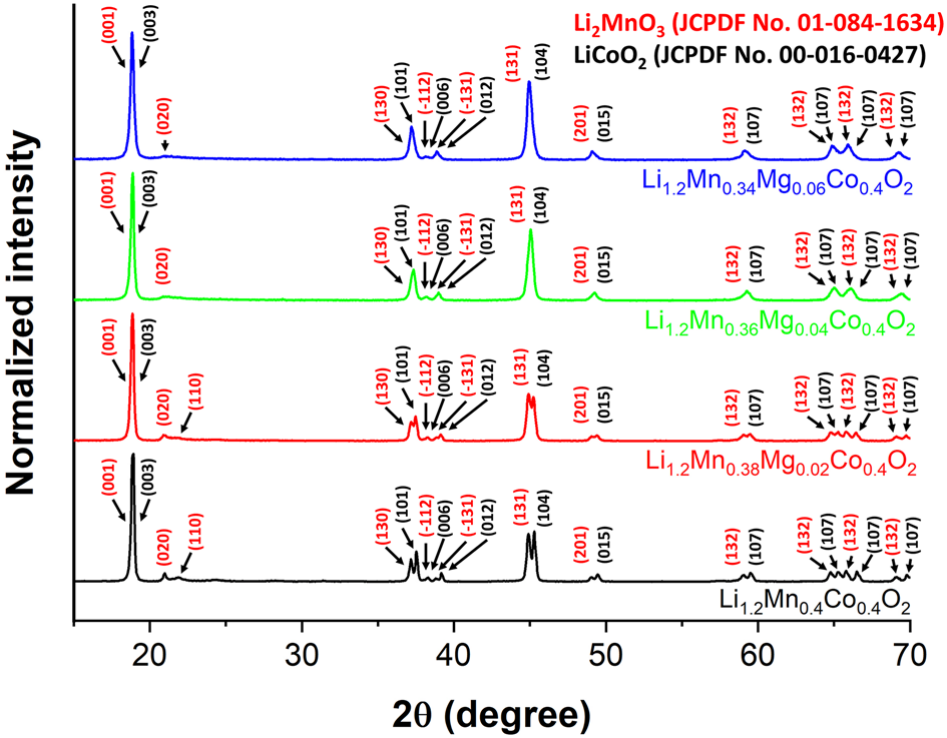
X-ray diffraction spectra of the pristine Li_1.2_Mn_0.4_Co_0.4_O_2_ and Mg-doped Li_1.2_Mn_0.4_Co_0.4_O_2_ materials.

X-ray diffraction (XRD) was employed to examine the crystal structure of the synthesized cathode materials. The results are presented in Fig. 2. The X-ray diffraction patterns of the electrode materials correspond to those of both monoclinic Li_2_MnO_3_ within space group $C2/m$ and rhombohedral LiCoO_2_ within space group $R\overline{3 }m$ with the structural characteristics of layered α-NaFeO_2_. Furthermore, the undoped material (Li_1.2_Mn_0.4_Co_0.4_O_2_) revealed diffraction peaks corresponding to Li_2_MnO_3_ and LiCoO_2_ phases that are clearly separate at 2θ positions above 35°. For the Mg-doped materials (Li_1.2_Mn_0.4-x_ Mg_x_Co_0.4_O_2_ (x = 0.02, 0.04, and 0.06)), decreased XRD peak separation was observed that depended upon Mg content. The doped materials with higher Mn contents, including Li_1.2_Mn_0.36_Mg_0.04_Co_0.4_O_2_ and Li_1.2_Mn_0.34_Mg_0.06_Co_0.4_O_2_, showed well merged XRD peaks of the Li_2_MnO_3_ and LiCoO_2_ phases, as presented in Fig. S4. This reflects that Mg doping has a significant effect on the crystal structure of as-prepared cathode materials. Mg doping can increase the degree of mixing between Li_2_MnO_3_ and LiCoO_2_ phases. This was especially observed in Li_1.2_Mn_0.36_Mg_0.04_Co_0.4_O_2_ and Li_1.2_Mn_0.34_Mg_0.06_Co_0.4_O_2_ materials. Doping with Mg^2+^ can significantly reduce the mixing energy between the Li_2_MnO_3_ and LiCoO_2_ phases, leading to a higher degree of mixing between these phases compared with an undoped cathode material. This phenomenon was confirmed by high-resolution transmission electron microscopy (HRTEM) in Fig. 1. Moreover, Rietveld refinement was performed to study the influences of Mg doping on the structural properties of Li_1.2_Mn_0.4_Co_0.4_O_2_ electrodes, as demonstrated in Fig. S5. Information about related atomic positions is given in Table S2. The calculated lattice constants are presented in Table S3. These results illustrate that the unit cell volume decreased as Mg content increased, as shown in Fig. S6a. Additionally, β was also reduced when the level of Mg doping increased, as presented in Fig. S6b. This indicates that Mg doping significantly induced structural distortion in Li_1.2_Mn_0.4_Co_0.4_O_2_ materials. XRD results show more broad and merged peaks when the Mg content is increased. This may introduce inaccuracies into XRD analysis of the Mg position. Thus, X-ray absorption spectroscopy, which is sensitive to the local atomic structure of materials, was used to investigate the Mg position in Mg-doped materials.

**Figure 3:**
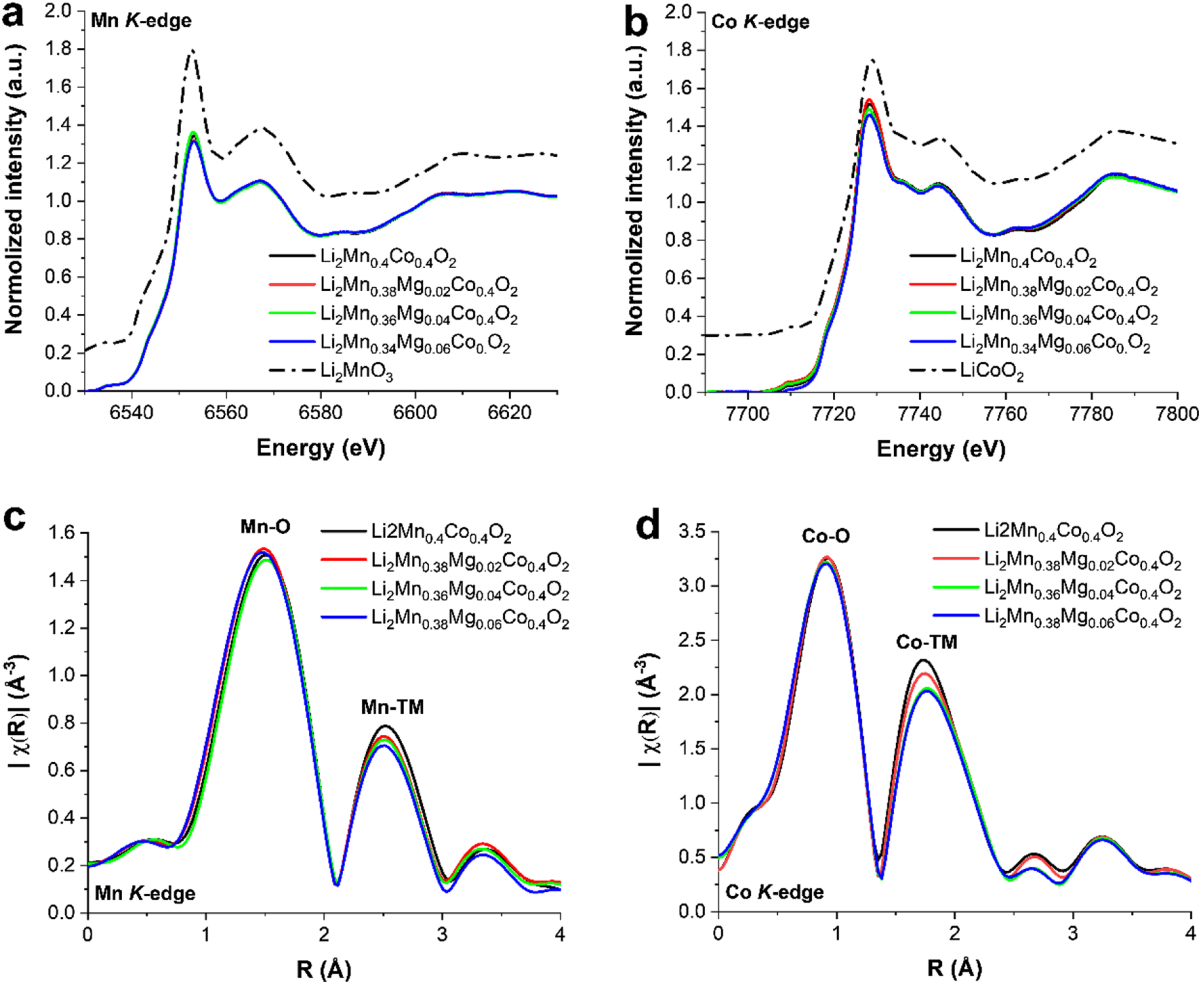
XANES profiles and *k*^2^-weighted Fourier-transformed EXAFS signals at the Mn (**a** and **c**) and Co (**b** and **d**) *K*-edges of pristine Li_1.2_Mn_0.4_Co_0.4_O_2_, Mg-doped Li_1.2_Mn_0.4_Co_0.4_O_2,_ Li_2_MnO_3_, and LiCoO_2_ materials.

**Figure 4:**
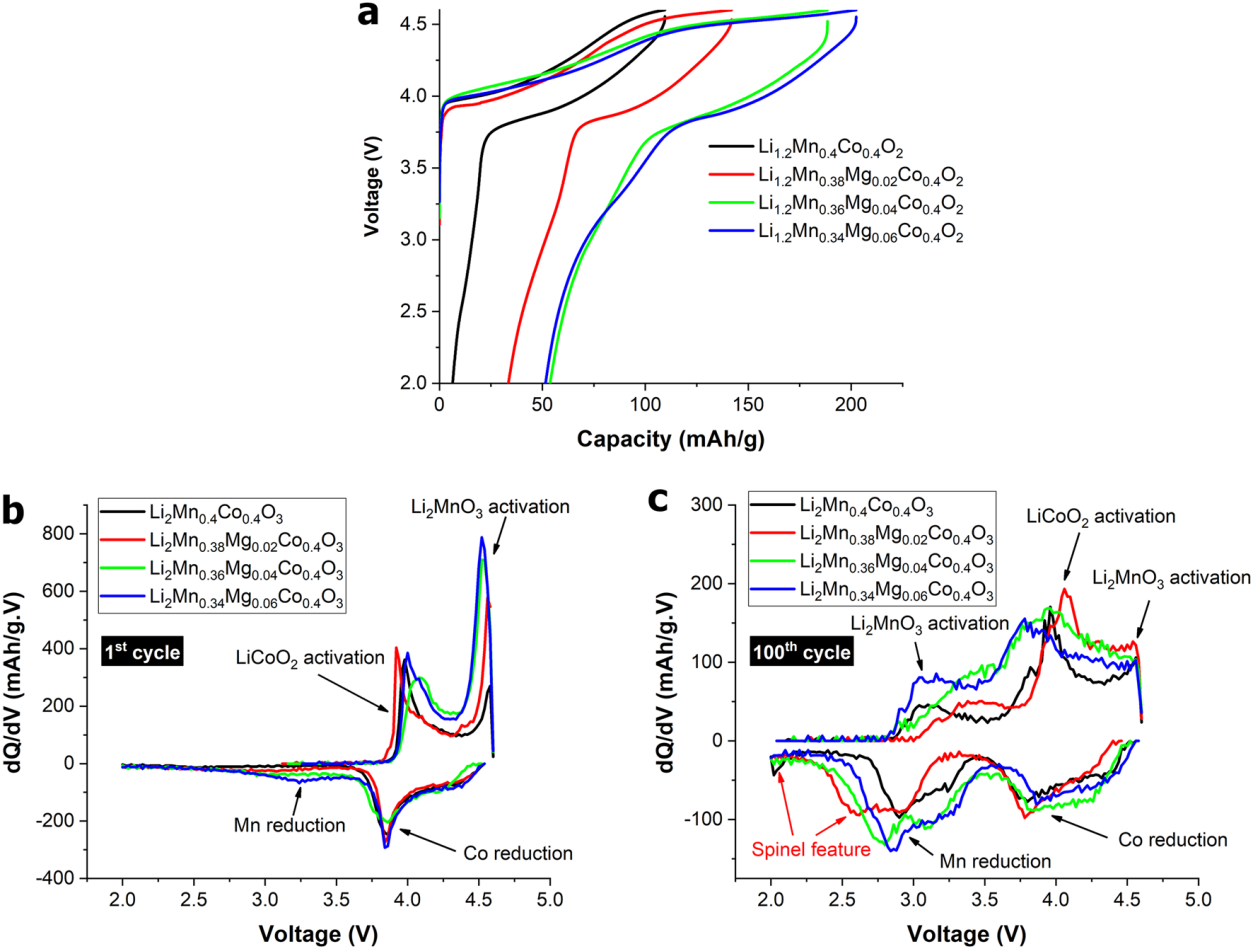
Voltage profiles (**a**), differential capacity plots at the 1st cycle (**b**), and differential capacity plots at the 100th cycle (**c**) of pristine Li_1.2_Mn_0.4_Co_0.4_O_2_ and Mg-doped Li_1.2_Mn_0.4_Co_0.4_O_2_ materials cycled at C/3.

An XAS technique was employed to study the local atomic structure of pristine Li_1.2_Mn_0.4_Co_0.4_O_2_ and Mg-doped Li_1.2_Mn_0.4_Co_0.4_O_2_ materials. Figure 3 illustrates the XANES spectra at Mn and Co *K*-edges of the prepared cathodes compared with the XANES spectra of Li_2_MnO_3_ and LiCoO_2_ phases as references. It is found that the XANES features of the obtained cathodes are almost identical to those of Li_2_MnO_3_ and LiCoO_2_ phases at the Mn and Co *K*-edges, respectively. This can be ascribed to the composite behavior of the synthesized cathode materials. The synthesized cathodes consist of ${\text{Li}}_{{2}} {\text{MnO}}_{{{3}^{ - } }}$ and LiCoO_2_-like regions, corresponding to the HRTEM and XRD results. The oxidation states of the Mn and Co species are directly determined by the main absorption edge positions. At Mn and Co *K*-edges, undoped Li_1.2_Mn_0.4_Co_0.4_O_2_ and Mg-doped Li_1.2_Mn_0.4_Co_0.4_O_2_ materials showed primary absorption edges at the same position, confirmed by 1st derivatives of XAS spectra, as illustrated in Fig. S7. This indicates that Mn and Co atoms in these materials have the same valance states. Moreover, the XANES profiles are identical to those in previous reports using XAS techniques to examine the same cathode material type. Mn and Co atoms possess average valance states of 4 + and 3 + , respectively^34–36^. The XANES spectra at both the Mn and Co edges of pristine undoped Li_1.2_Mn_0.4_Co_0.4_O_2_ and Mg-doped Li_1.2_Mn_0.4_Co_0.4_O_2_ materials largely overlapped with small differences between them. This suggests that Mn and Co atoms in the materials are in similar environments with similar local atomic structures and slightly different structural distortion. Additionally, Figs. 3c and 4d reveal *k*^2^-weighted Fourier-transformed EXAFS signals at the Mn and Co *K*-edges of pristine Li_1.2_Mn_0.4_Co_0.4_O_2_ and Mg-doped Li_1.2_Mn_0.4_Co_0.4_O_2,_ materials, respectively. The first main peaks occurred because the interactions between Mn and Co adsorbing atoms, located in the octahedral sites, were surrounded by six oxygen atoms. The peaks are denoted as Mn–O and Co–O for the Mn and Co *K-*edges, respectively. The second peaks correspond to the interactions between Mn and Co adsorbing atoms and transition metal (TM) atoms in the transition metal layers. The peaks are denoted as Mn-TM and Co-TM for the Mn and Co *K-*edges, respectively. It is well established that the amplitudes of the Mn-TM and Co-TM peaks are highly sensitive to surrounding elements. Our previous work studying the same material type reported that Li_1.2_Mn_0.4_Co_0.4_O_2_ materials with various Li_2_MnO_3_ and LiCoO_2_ domain sizes provided different intensities of the Mn-TM and Co-TM peaks. This is occurred because Mn and Co are surrounded by different types and numbers of neighboring atoms that have different X-ray scattering capabilities^32^. In the current study, at the Co-*K* edge, the amplitude of Co-TM peak is decreased with the Li_2_MnO_3_ and LiCoO_2_ domain size (Mn content increased). This is because the Li_2_MnO_3_ and LiCoO_2_ phases are more distributed in the material with smaller Li_2_MnO_3_ and LiCoO_2_ domain sizes, while the individual Li_2_MnO_3_ and LiCoO_2_ phases are more locally isolated in the material with larger Li_2_MnO_3_ and LiCoO_2_ domain sizes. The result is that Co atoms in the material with smaller Li_2_MnO_3_ and LiCoO_2_ domain sizes interact with a larger number of Mn atoms (lighter atoms). There is less scattering than Co and consequently a lower amplitude Co-TM peak is produced. At the Mn-*K* edge, our previous report^32^ presented that a Li_1.2_Mn_0.4_Co_0.4_O_2,_ material with a smaller Li_2_MnO_3_ domain size exhibited a stronger intensity of the Mn-TM peak. This is occurred because the Mn atoms are surrounded by a larger Co species, leading to better scattering than for Mn alone and increased amplitude of the Mn-TM peak. In contrast, the current study showed that Mg-doped cathode materials reveal decreased amplitude of Mn-TM although Li_2_MnO_3_ and LiCoO_2_ domain sizes decreased (Mn content increased). This results from the presence of Mg in transition metal layers, which has a very low x-ray scattering power compared with Mn and Co species. So, the intensity of the Mn-TM peak decreased as the Mg content increased. The XAS experimental results revealed that the domain sizes of Li_2_MnO_3_ and LiCoO_2_ phases decreased when the Mg content increased, which is in complete agreement with the XRD and HRTEM results. Additionally, the decreased amplitude of Mn-TM is evidence of the presence of Mg in Mn-sites in the transition metal layers of the Li_2_MnO_3_ structure. Moreover, EXAFS fitting was performed to calculate the chemical bond lengths of Mn–O, Mn-TM, Co–O, and Co-TM to obtain deeper information involving the impacts of Mg doping on the local structure of Mg-doped Li_1.2_Mn_0.4_Co_0.4_O_2_ materials. Fourier-transformed EXAFS signals with fitted spectra at the Mn and Co *K*-edges are given in Figs. S8 and S9, respectively. The calculated bond distances and agreement indices are presented in Table S4. These results imply that Mg doping significantly decreases the Mn–O bond length with increasing Mg content, as can be seen in Fig. S10. This suggests that Mg doping can probably strengthen the chemical bonds and reinforce interactions of MnO_6_-octahedral slabs, leading to improved structural stability^37^.

### Electrochemical characterization

**Figure 5:**
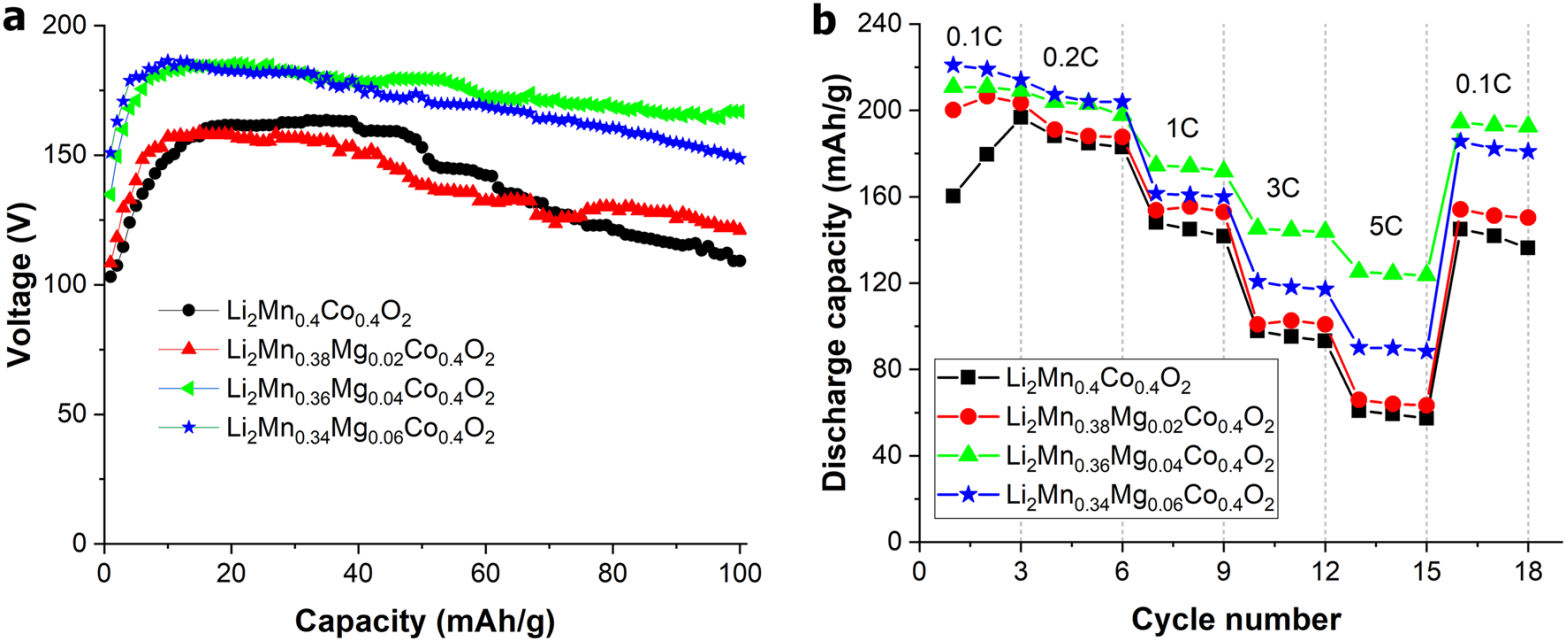
Cycling stability (**a**) and rate performance (**b**) of pristine Li_1.2_Mn_0.4_Co_0.4_O_2_ and Mg-doped Li_1.2_Mn_0.4_Co_0.4_O_2_ materials.

The electrochemical performance of the prepared electrodes was examined using galvanostatic cycling, as illustrated in Fig. S11. Figure 4a shows the voltage profiles obtained during the initial cycle. The voltage profiles of the electrode consist of two notable plateaus at around 3.9 V and 4.4 V, corresponding to activation of the LiCoO_2_ and Li_2_MnO_3_ components, respectively. For composite-based Li- and Mn-rich layered oxide cathodes, it is established that the Li_2_MnO_3_ phase has an important role in storing excess lithium for stabilizing the LiMO_2_ component (M=Co, Ni, and Mn), bringing about improved structural stability during electrochemical cycling^18,31,38,39^. The electrochemical mechanism of the prepared cathode materials can be explained as follows. During the initial charging process at around 3.9 V to 4.4 V, Li ions are extracted from the LiCoO_2_ structure to form a CoO_2_ phase, resulting in the oxidation of Co^3+^ to Co^4+^, while the Li_2_MnO_3_ component is electrochemically inactive. This causes depletion of Li ions in the lithium layers of the LiCoO_2_ structure. Li ions from octahedral sites in the Li and Mn layers of the Li_2_MnO_3_ component subsequently diffuse into the lithium-depleted layers of the LiCoO_2_ structure to maintain structural stability^40,41^. For the second voltage plateau, at about 4.4 V to 4.6, Li ions are extracted from the Li_2_MnO_3_ structure to produce Li_2_O and layered MnO_2_ phases. Formation of a Li_2_O phase results in irreversible capacity loss in the initial cycle^31,38^. During the discharge process, a Li ion is inserted into the Li_1−x_CoO_2_ structure at around 4.6–3.8 V, while a Li ion is inserted into MnO_2_ phases below 3.8 V, corresponding to a reduction of Mn^4+^ to Mn^3+^ to form a layered LiMnO_2_ phase. It is established that the formed LiMnO_2_ phase always experiences transition from a layered to a spinel-like structure upon electrochemical cycling, which subsequently induces a large capacity loss and voltage drop during repeated cycling. This indicates that the activation of Li_2_MnO_3_ to form a LiMnO_2_ phase can induce a spinel-like phase transition during cycling. Previous reports revealed that the degree of Li_2_MnO_3_ activation is largely determined by the Li_2_MnO_3_ domain size and current rate^33,42,43^. A small Li_2_MnO_3_ domain size and low current rate could facilitate activation of the Li_2_MnO_3_ phase and subsequently cause a large initial irreversible capacity and a spinel-like phase transition, leading to poor cyclability. This is shown in the electrochemical results observed in the initial voltage profiles of the prepared electrodes, given in Fig. 4a. The initial irreversible capacities of Li_1.2_Mn_0.4_Co_0.4_O_2_, Li_1.2_Mn_0.38_Mg_0.02_Co_0.4_O_2_, Li_1.2_Mn_0.36_Mg_0.04_Co_0.4_O_2_, and Li_1.2_Mn_0.34_Mg_0.06_Co_0.4_O_2_ cathodes are 6.39 mAh/g, 33.46 mAh/g, 53.67 mAh/g, and 51.47 mAh/g, respectively. This indicates that an electrode with a smaller Li_2_MnO_3_ domain size can allow a larger level of Li_2_MnO_3_ activation, which is directly related to the range of the voltage plateau at around 4.4 V. A larger activation brings about higher initial irreversible capacity. This is because a smaller Li_2_MnO_3_ domain size is easier to activate. Figure 4b shows differential capacity plots at the 1st cycle of the electrode materials. The oxidation peaks at around 3.9 V and 4.5 V result from LiCoO_2_ and Li_2_MnO_3_ activation, respectively. The degree of Li_2_MnO_3_ activation is directly correlated with the intensity of the oxidation peak at 4.5 V. Mg-doped cathodes reveal a higher oxidation peak intensity at 4.5 V than undoped cathodes. This is because the cathodes with smaller Li_2_MnO_3_ domain sizes induce greater Li_2_MnO_3_ activation, especially for Li_1.2_Mn_0.36_Mg_0.04_Co_0.4_O_2_, and Li_1.2_Mn_0.34_Mg_0.06_Co_0.4_O_2_ cathodes. Figure 4c presents differential capacity plots after the 100th cycle for the electrode materials. The results show that the oxidation peak at 4.5 V almost disappears because most of the Li_2_MnO_3_ component has been activated to form a LiMnO_2_ phase. The reduction peaks at about 3.9 V and 2.8 V correspond to Li-ion insertion into the CoO_2_ and MnO_2_ phases to form LiCoO_2_ and LiMnO_2_, respectively. An undoped cathode (Li_1.2_Mn_0.4_Co_0.4_O_2_) shows a small oxidation peak at 2.0 V, corresponding to spinel-like phase features^33^. Furthermore, a Mg-doped cathode with a low Mn content (Li_1.2_Mn_0.38_Mg_0.02_Co_0.4_O_2_) reveals a shift of the reduction peak at 2.8 V to a lower voltage of around 2.5 V, indicating that the cathode exhibits a spinel-like phase evolution during electrochemical cycling^44–46^. In contrast, the Li_1.2_Mn_0.36_Mg_0.04_Co_0.4_O_2_ and Li_1.2_Mn_0.34_Mg_0.06_Co_0.4_O_2_ cathodes reveal a smaller degree of peak shift toward lower voltages. This indicates that doping a Li_1.2_Mn_0.4_Co_0.4_O_2_ cathode with an appropriate Mg content can help to suppress spinel-like phase transition during cycling. According to XAS experimental results, for Mg-doped cathodes, Mg doping decreases the bond length between Mn and O (Mn–O), suggesting that Mg substitution strengthens the chemical bonds and reinforces interactions of the MnO_6_-octahedral slabs, leading to enhanced structural stability. Additionally, this suggests that the level of structural transition highly depends on Mg content. Figure 5a shows that the Li_1.2_Mn_0.36_Mg_0.04_Co_0.4_O_2_ and Li_1.2_Mn_0.34_Mg_0.06_Co_0.4_O_2_ cathodes have higher reversible capacity than other cathodes. This phenomenon results from a smaller Li_2_MnO_3_ domain size that causes a larger level of Li_2_MnO_3_ to form a greater amount of the electrochemically active MnO_2_ phase, leading to higher reversible capacity. Additionally, the Li_1.2_Mn_0.36_Mg_0.04_Co_0.4_O_2_ cathode has higher cycling stability than other candidate cathodes. This indicates that Li_1.2_Mn_0.36_Mg_0.04_Co_0.4_O_2_ is an optimal Mn doping (x = 0.04) condition, while cathodes with higher Mg doping (Li_1.2_Mn_0.34_Mg_0.06_Co_0.4_O_2_, x = 0.06) show lower cycling stability than the Li_1.2_Mn_0.36_Mg_0.04_Co_0.4_O_2_ cathode_._ Doping with a very high Mg content (x = 0.06) can induce a larger structural distortion, corresponding to severe decrease in the β value observed in Rietveld refinement results, as shown in Fig. S6b. Greater structural distortion can lead to poorer structural stability, producing cathodes with lower cyclability than the Li_1.2_Mn_0.36_Mg_0.04_Co_0.4_O_2_ cathode. Moreover, the Li_1.2_Mn_0.36_Mg_0.04_Co_0.4_O_2_ cathode also has noticeably better rate capability than other candidate cathodes, as revealed in Fig. 5b, since Li_2_MnO_3_ is electrochemically inert. Therefore, Li_1.2_Mn_0.4_Co_0.4_O_2_ and Li_1.2_Mn_0.38_Mg_0.02_Co_0.4_O_2_ cathodes with a larger Li_2_MnO_3_ domain size often exhibit lower reversible capacity and poorer rate performance. Additionally, Li_1.2_Mn_0.4_Co_0.4_O_2_ and Li_1.2_Mn_0.38_Mg_0.02_Co_0.4_O_2_ cathodes also exhibit a large structural transition during cycling. This structural transition can lead to severe structural disorder and larger defects. For Li_1.2_Mn_0.34_Mg_0.06_Co_0.4_O_2_ at higher Mg doping, the cathode consists of the smallest Li_2_MnO_3_ domain size but has poor structural stability. This leads to structural degradation and defect formation during cycling, which hinders Li-ion transport in the electrode. These phenomena act as barriers for Li-ion mobility in cathode materials that consequently result in poorer rate performance. This gives the Li_1.2_Mn_0.36_Mg_0.04_Co_0.4_O_2_ cathode a smaller Li_2_MnO_3_ domain size and higher structural stability during electrochemical cycling, presenting higher rate capability than the other cathode materials. Interestingly, this is contrary to our previous works reporting that an undoped Li_1.2_Mn_0.36_Mg_0.04_Co_0.4_O_2_ cathode with a smaller Li_2_MnO_3_ domain size had lower cycling stability that of the cathode with a larger Li_2_MnO_3_ domain size. This is because a small Li_2_MnO_3_ domain was easily activated and further transformed to a spinel-like phase, subsequently leading to more severe structural deterioration upon cycling^30,32^. It can be confirmed that Mg doping enhances structural stability of a cathode material.

Electrochemical impedance spectroscopy (EIS) was done to examine the electronic conductivity of the cathodes after 100 cycles, as shown in Fig. S12. The calculated charge transfer resistance (R_CT_) is shown in Table S5. For fresh cells, Li_1.2_Mn_0.4_Co_0.4_O_2_, Li_1.2_Mn_0.38_Mg_0.02_Co_0.4_O_2_, Li_1.2_Mn_0.36_Mg_0.04_Co_0.4_O_2_, and Li_1.2_Mn_0.34_Mg_0.06_Co_0.4_O_2_ cathodes revealed R_CT_ values of 9.235 Ω, 8.452 Ω, 8.324 Ω, and 8.624 Ω, respectively. After 100 cycles, the R_CT_ values were respectively 739.485Ω, 345.214 Ω, 305.236 Ω, and 479.258 Ω for these cathodes. Li_1.2_Mn_0.36_Mg_0.04_Co_0.4_O_2_ exhibited the lowest R_CT_ among the cathodes after repeated cycling. This indicates that the other cathodes had a higher level of structural transition and degradation during electrochemical cycling, generating a greater degree of structural disorder and defects. These phenomena have negative impacts on both the ionic and electronic conductivities of the cathode materials.

The electrochemical cycling process of a Li-ion battery can be explained as Li-ion insertion and extraction in the structures of its electrode and cathode materials. This indicates that understanding the kinetic parameters impacting Li-ion diffusion coefficients of electrode materials is important to enable design and develop electrode materials for Li-ion batteries with good electrochemical performance. A galvanostatic intermittent titration technique (GITT) is widely used to investigate the lithium-ion mobility behaviors of electrode materials. The lithium-ion diffusion coefficient (*D*_*Li*_^+^) can be expressed by Fick's Second Law of Diffusion, as demonstrated in Eq. 1^47,48^:

1 $${\varvec{D}}_{{Li^{ + } }} = \frac{{\mathbf{4}}}{{\varvec{\pi}}}\left( {\frac{{{\varvec{m}}_{{\varvec{B}}} {\varvec{V}}_{{\varvec{M}}} }}{{{\varvec{M}}_{{\varvec{B}}} {\varvec{S}}}}} \right)^{{\mathbf{2}}} \left( {\frac{{\Delta E_{S} }}{{{\varvec{\tau}}\left( {\frac{{{\text{d}}E_{{\varvec{\tau}}} }}{{{\varvec{d}}\sqrt {\varvec{\tau}} }}} \right)}}} \right)^{2} \;\;\left( {\tau < < \frac{{{\varvec{L}}^{2} }}{{{\varvec{D}}_{{Li^{ + } }} }}} \right)$$

where ${{\varvec{m}}}_{{\varvec{B}}}$ and ${{\varvec{M}}}_{{\varvec{B}}}$ are the molecular weight and mass of the active material, respectively. ${{\varvec{V}}}_{{\varvec{m}}}$ is the molar volume of the active material. ${\varvec{S}}$ is defined as the surface area of the electrode. *L* is electrode thickness. Figure S13 reveals that the relationship between the cell voltage profile (E) and t^1/2^ is nearly linear. Consequently, Eq. (1) can be simplified as^49,50^:

2 $${\varvec{D}}_{{Li^{ + } }} = \frac{{\mathbf{4}}}{{\user2{\pi \tau }}}\left( {\frac{{{\varvec{m}}_{{\varvec{B}}} {\varvec{V}}_{{\varvec{M}}} }}{{{\varvec{M}}_{{\varvec{B}}} {\varvec{S}}}}} \right)^{{\mathbf{2}}} \left( {\frac{{\Delta E_{{\varvec{S}}} }}{{\Delta E_{{\varvec{\tau}}} }}} \right)^{{\mathbf{2}}} \;\;\left( {\tau < < \frac{{{\varvec{L}}^{2} }}{{{\varvec{D}}_{{Li^{ + } }} }}} \right)$$

**Figure 6:**
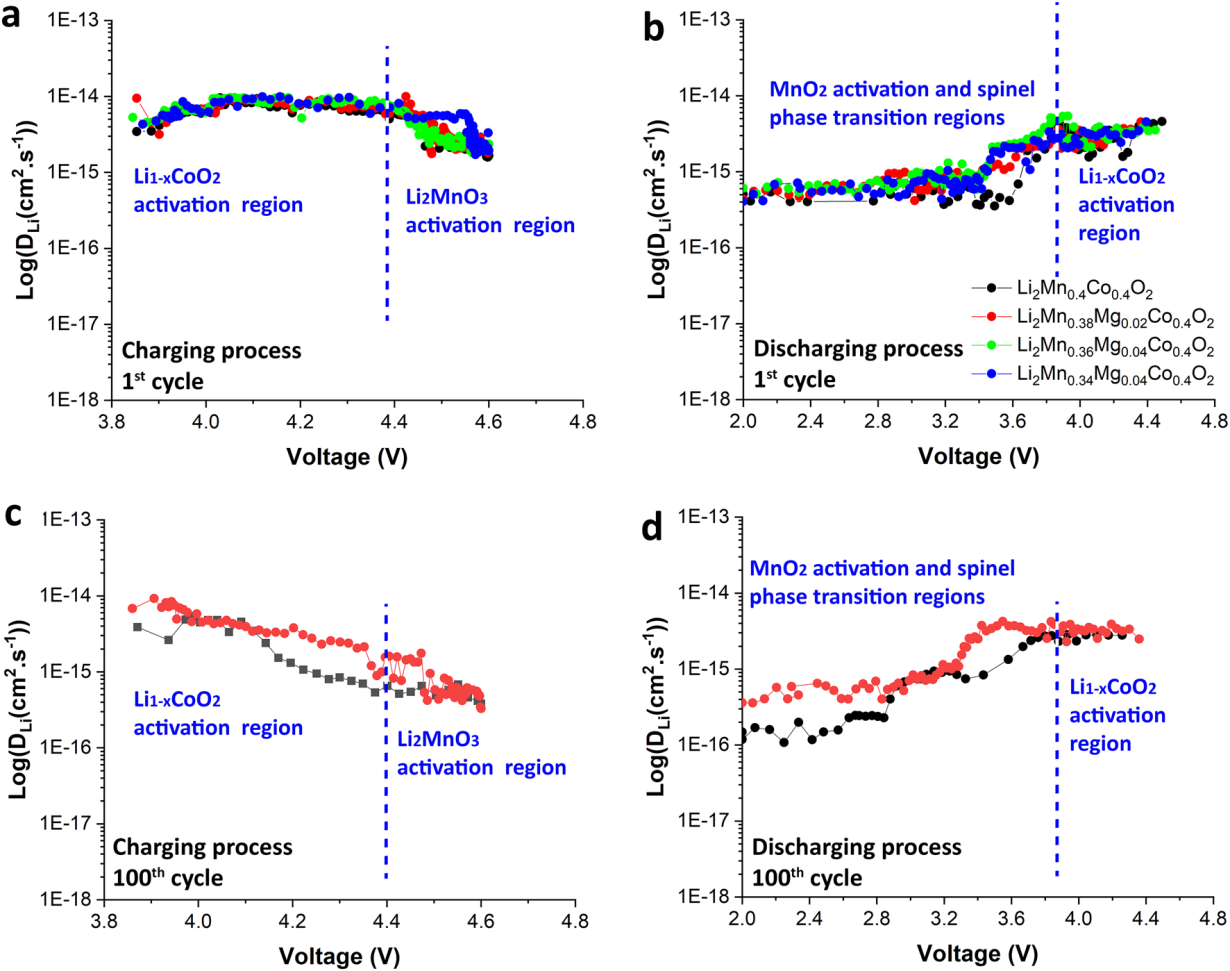
Calculated lithium-ion diffusion coefficients as a function of cell voltage at the 1st and 100th cycles during charging (**a** and **c**) and discharging (**b** and **d**) processes.

The electrochemical mechanisms of the Li and Mn rich layered oxide cathode material class are complex, involving lithium-ion diffusion, oxygen loss, and structural transformation. Therefore, this work emphasizes an investigation of the overall Li-ion diffusion coefficients of the prepared cathode materials. The calculated coefficients are defined as apparent Li-ion diffusion coefficients^51,52^. The GITT profiles and calculated lithium-ion diffusion coefficients as a function of time for Li_1.2_Mn_0.4_Co_0.4_O_2_ and Li_1.2_Mn_0.36_Mg_0.04_Co_0.4_O_2_ cathode materials at the first and 100th cycles are revealed in Fig. S14. Figure 6a and b illustrate calculated lithium-ion diffusion coefficients as a function of cell voltage for Li_1.2_Mn_0.4_Co_0.4_O_2_ and Mg-doped Li_1.2_Mn_0.4_Co_0.4_O_2_ electrodes. In Fig. 6a and 6b, the charging process exhibits two distinct lithium-ion diffusion regions. The calculated lithium-ion diffusion coefficient values of the prepared cathode are very similar. The first region corresponds to the extraction of Li ions from the LiCoO_2_ structure at voltages below 4.4 V. The calculated lithium-ion diffusion coefficients of the Li_1.2_Mn_0.36_Mg_0.04_Co_0.4_O_2_ electrode are slightly higher than those of the other electrodes. These calculated lithium-ion diffusion coefficients increased slightly from around 3.44 × 10^–15^ cm^2^ s^−1^ to 9.57 × 10^–15^ cm^2^ s^−1^ when the electrodes were charged to 4.04 V. Then, the coefficients decreased gradually from around 9.57 × 10^–15^ to 5.98 × 10^–15^ cm^2^ s^−1^ when the electrodes were charged to 4.40 V. The second region is ascribed to extraction of Li ions from the Li_2_MnO_3_ component at voltages above 4.4 V. The calculated lithium-ion diffusion coefficients decreased rapidly from around 5.98 × 10^–15^ cm^2^ s^−1^ to 1.66 × 10^–18^ cm^2^ s^−1^ when the electrodes were charged to 4.6 V. This reveals that the Li-ion diffusion coefficients of the Li_2_MnO_3_ component are lower than those of the LiCoO_2_ component due to the lower ionic conductivity of the Li_2_MnO_3_ component. For the initial discharge processes, the calculated lithium-ion diffusion coefficients of the Li_1.2_Mn_0.36_Mg_0.04_Co_0.4_O_2_ electrode are slightly higher than those of the candidate electrodes. Two lithium-ion diffusion regions were clearly observed. The first region corresponds to Li-ion intercalation into the Li_1−x_CoO_2_ structure at higher voltages. The second region occurred from Li-ion intercalation into MnO_2_ and a newly formed spinel phase at lower voltages. The Li-ion diffusion coefficients obtained from the Li_1-x_CoO_2_ activation region were higher than those of the MnO_2_ and spinel structures. This occurred because of structural disorder and defects produced during the spinel phase transformation. The results show that the lithium-ion diffusion coefficient slightly increased with the Mg content, but the Li_1.2_Mn_0.34_Mg_0.06_Co_0.4_O_2_ electrode with the highest Mg content shows lower a lithium-ion diffusion coefficient than Li_1.2_Mn_0.36_Mg_0.04_Co_0.4_O_2_ electrode because a higher level of Mg doping induces a larger structural distortion, obstructing lithium-ion transport in the structure. This causes the Mg-doped cathodes to exhibit slightly improved rate capability, especially for the Li_1.2_Mn_0.34_Mg_0.06_Co_0.4_O_2_ electrode, as demonstrated in Fig. 5b. Moreover, the calculated lithium-ion diffusion coefficients during charging and discharging processes of the prepared electrodes after 100 cycles are shown in Fig. 6c and d. The experimental results reveal that the Li_1.2_Mn_0.04_Co0_.4_O_2_ electrode exhibited lower Li-ion diffusion coefficients than those of the Li_1.2_Mn_0.36_Mg_0.04_Co_0.4_O_2_ electrode. The reason for this is that the Li_1.2_Mn_0.04_Co0_.4_O_2_ electrode encounters a greater level of the spinel phase transition that subsequently results in more structural disorder and larger defects. This leads to severe structural degradation that hinders Li-ion diffusion, inducing slower Li-ion transport in the Li_1.2_Mn_0.04_Co0_.4_O_2_ cathode. These results imply that appropriate Mg doping (x = 0.04) can effectively help to stabilize the structure of Li- and Mn-rich layered oxide cathode materials. This gives the Li_1.2_Mn_0.36_Mg_0.04_Co_0.4_O_2_ cathode material a higher cycling stability and noticeably better rate capability than other candidate cathodes.

**Figure 7:**
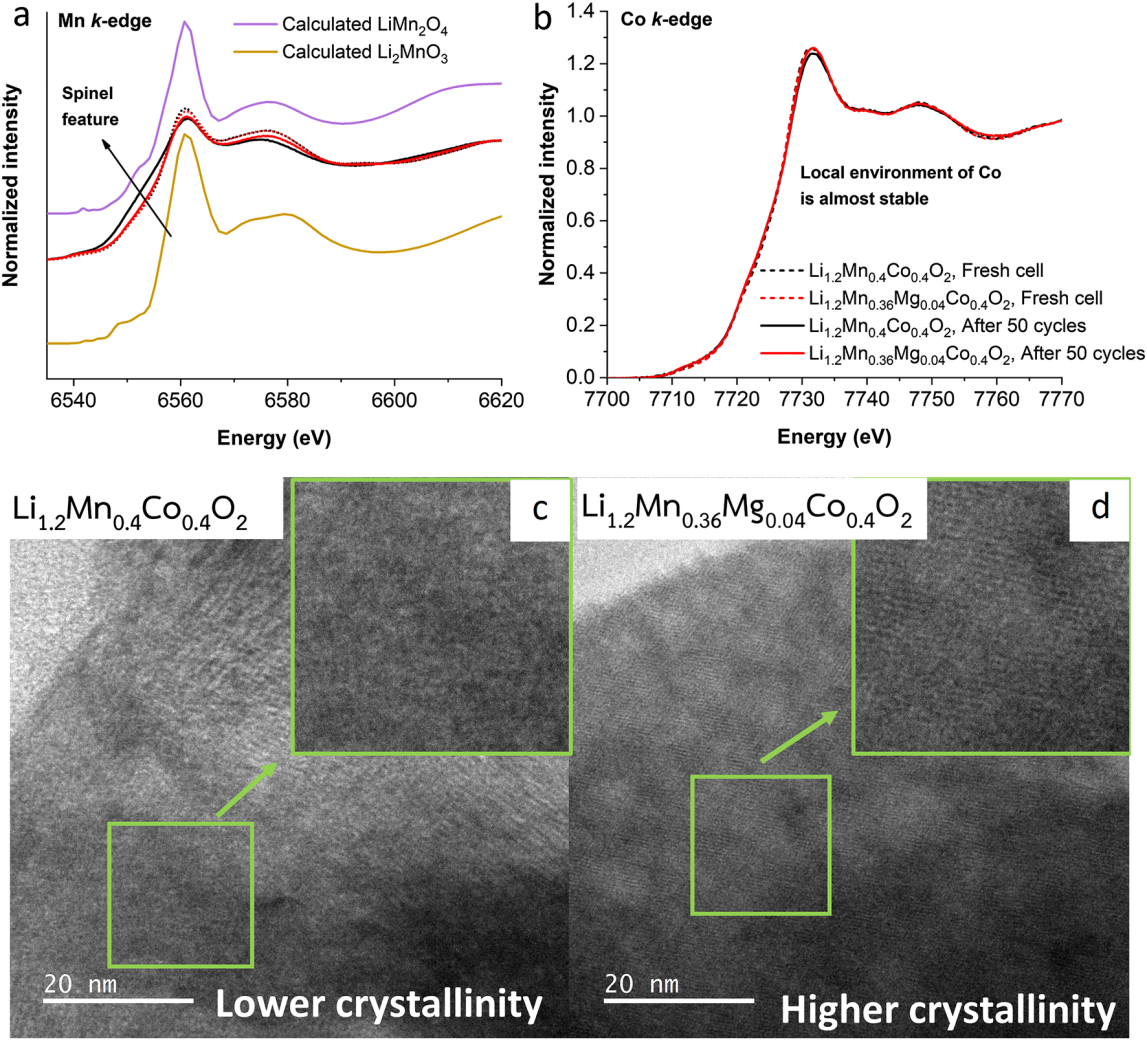
XANES spectra at Mn (**a**) and Co (**b**) *K*-edges of a fresh cell and cells cycled at various current rates after 50 cycles, HRTEM images of cycled Li_1.2_Mn_0.4_Co_0.4_O_2_ (**c**) and Li_1.2_Mn_0.36_Mg_0.04_Co_0.4_O_2_ (**d**) electrodes after 100 cycles.

XAS and HRTEM techniques were employed to examine the structural evolution of the Li_1.2_Mn_0.4_Co_0.4_O_2_ and Li_1.2_Mn_0.36_Mg_0.04_Co_0.4_O_2_ cathodes after prolonged cycling. Figure 7a and b illustrate calculated and measured XANES spectra of a pristine electrode and cycled electrodes after 50 cycles for Mn and Co *K*-edges, respectively. For the Mn *K*-edge, experimental results show that the local environment around the Mn atoms in the Li_1.2_Mn_0.4_Co_0.4_O_2_ cathode changed significantly, while that of Li_1.2_Mn_0.36_Mg_0.04_Co_0.4_O_2_ cathodes slightly changed compared to the fresh cathodes. Especially in the case of Li_1.2_Mn_0.4_Co_0.4_O_2_ cathode, the rising-edge feature of the electrode was closer to the rising edge of a spinel LiMn_2_O_4_ phase than that of a Li_1.2_Mn_0.4_Co_0.4_O_2_ cathode. This indicates that proper Mg doping can retard structural transitions during electrochemical cycling. Additionally, for the Co *K*-edge, the local environment around the Co atoms was quite stable. However, a Li_1.2_Mn_0.4_Co_0.4_O_2_ cathode with a larger spinel phase evolution exhibited slight local environmental changes. HRTEM images of the Li_1.2_Mn_0.4_Co_0.4_O_2_ and Li_1.2_Mn_0.36_Mg_0.04_Co_0.4_O_2_ cathodes were used to present the degree of overall structural degradation after 100 cycles, as seen in Fig. 7c and d. The Li_1.2_Mn_0.4_Co_0.4_O_2_ cathode exhibits lower crystallinity than the Li_1.2_Mn_0.36_Mg_0.04_Co_0.4_O_2_ cathode. This results from a lager phase transition leading to more severe structural deterioration. It reflects that Mg doping can improve the overall structural stability of the Li- and Mn-layered oxide cathode material class upon cycling.

**Figure 8:**
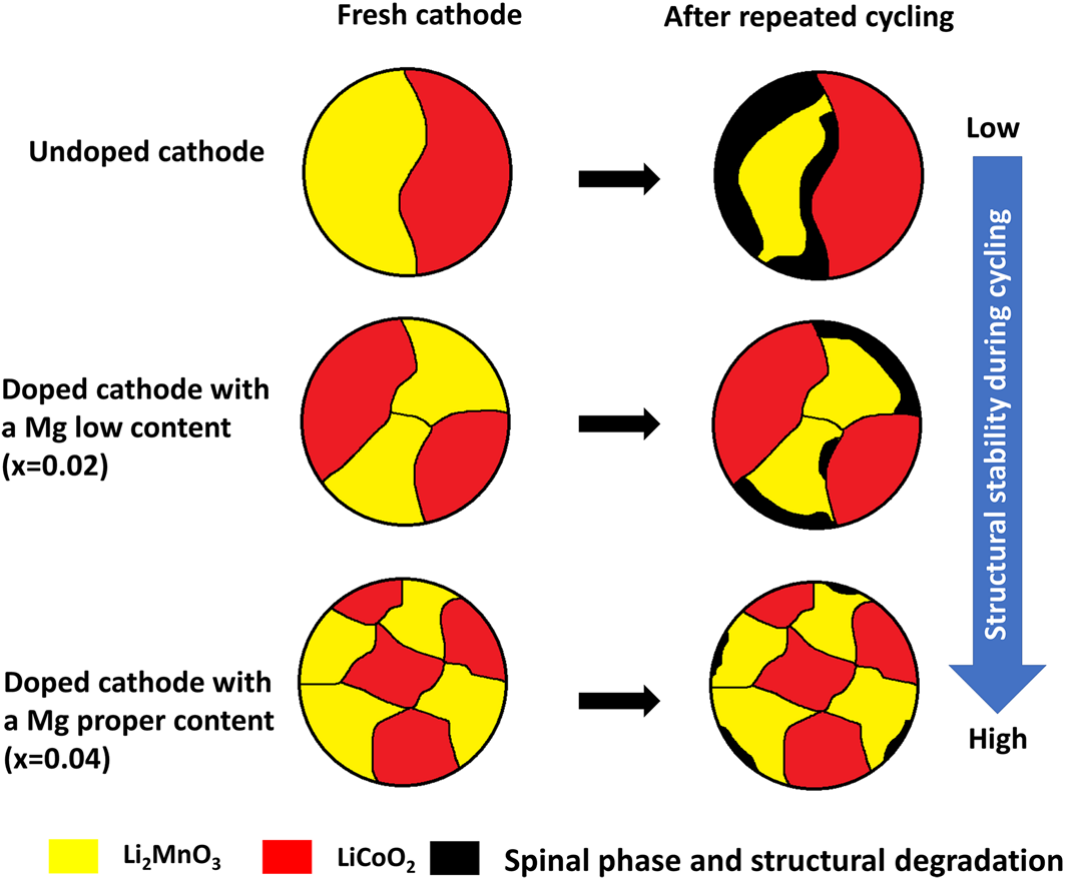
Qualitative illustration of degradation due to structural transition of cathode materials with various Mg dopant levels.

This work found that the prepared cathodes were of a composite material type consisting of Li_2_MnO_3_ and LiCoO_2_ components, which was confirmed from HRTEM, XRD, and XAS results. Li_2_MnO_3_ and LiCoO_2_ domain sizes decreased as the Mg content increased. The XRD results show more broad and merged peaks when the Mg content is increased. This may introduce inaccuracies into XRD analysis of the Mg position. Thus, X-ray absorption spectroscopy, which is sensitive to the local atomic structure of materials, was used to investigate the Mg position in Mg-doped materials. Various Li_2_MnO_3_ sizes play a critical role in the electrochemical behaviors of the prepared materials. This is because Li_2_MnO_3_, with a smaller domain size, can be easily activated, leading to higher reversible capacity. However, activation of the Li_2_MnO_3_ component always results in transition from a layered to a spinel-like structure upon electrochemical cycling. This brings about structural degradation and consequently poor electrochemical performance. Additionally, EDS and XAS results imply that Mg was successfully doped into Mn sites in the structure of the Li_2_MnO_3_ component. The electrochemical results reveal that appropriate Mg doping can improve the structural stability of a cathode material, leading to enhanced electrochemical properties. The impacts of Mg doping on the microstructure and structural degradation mechanisms during repeated cycling of a composite-based Li-and Mn-rich layered oxide cathode material are displayed in Fig. 8. Li_1.2_Mn_0.36_Mg_0.04_Co_0.4_O_2_ with small Li_2_MnO_3_ domain sizes exhibited improved electrochemical performance. In this study, the electrochemical performance of the synthesized cathode materials is lower than those of Li-and Mn-rich layered oxide cathode materials in previous work. The obtained information reveals deeper understanding about the roles of Mg doping on the structural characteristics, electrochemical properties, and structural degradation mechanisms of Li-and Mn-rich layered oxide cathode materials. This provides an essential guideline for structural design and stabilization of composite-based cathode materials for lithium-ion batteries.

## Conclusions

Li_1.2_Mn_0.4_Co_0.4_O_2_ and Li_1.2_Mn_0.4-x_ Mg_x_Co_0.4_O_2_ (x = 0.00, 0.02, 0.04, and 0.06) cathodes were successfully prepared using a sol–gel method. Mg doping significantly affects the microstructure, crystal structure, and local atomic structure of the prepared cathode materials. The cathodes were composite-based materials, exhibiting different phase separation behaviors of their Li_2_MnO_3_ and LiCoO_2_ components. The Li_2_MnO_3_ and LiCoO_2_ domain sizes both decreased as the Mg content increased, significantly affecting the electrochemical behaviors of the prepared materials. The level of phase transition occurring during cycling also depends on the Mg content. A Li_1.2_Mn_0.36_ Mg_0.04_Co_0.4_O_2_ cathode material with a small Li_2_MnO_3_ domain size and appropriate Mn doping (x = 0.04) provided higher reversible capacity, improved cycling stability, and better rate capability. Mg doping can stabilize the structure of a cathode. This occurs because Mg doping increases Mn–O chemical bonding and reinforces interactions of MnO_6_-octahedral slabs, which subsequently leads to improved structural stability by retarding structural transformation and degradation during cycling. This work provides a better understanding about the effects of Mg doping on the structural characteristics and degradation mechanisms during cycling. It provides useful information for designing the structure and enhancing the structural stability of composite-based cathode materials for lithium-ion batteries.

## Methods

### Sample preparation

Li_1.2_Mn_0.4-x_ Mg_x_Co_0.4_O_2_ (x = 0.00, 0.02, 0.04, and 0.06) cathode materials were synthesized using a sol–gel method. Li(CH_3_COO)·2H_2_O (Aldrich), Mn(CH_3_COO)_2_·4H_2_O(Aldrich), Co(CH_3_COO)_2_·4H_2_O, and Mg(CH_3_COO)_2_·4H_2_O (Q RëC™) were used as starting materials. Citric acid was employed as a chelating agent with a molar ratio of metal ions to citric acid of 2:1. First, the required amounts of the raw materials were dissolved in deionized water while citric acid was separately dissolved in deionized water. Then, the aqueous citric acid solution was slowly added into the aqueous precursor solution. After that, the mixed solution was vigorously stirred at 80 °C until a gel formed. This gel was dried at 100 °C for 12 h and then pre-heated at 450 °C for 5 h to remove organic compounds. Finally, the obtained mixture was heated at 800 °C in air for 10 h and naturally cooled to room temperature in a box furnace.

### Structural and morphological characterization

The local crystal and atomic structures of the prepared electrodes were examined using X-ray diffraction (XRD) (RIGAKU TTRAX III) and X-ray absorption spectroscopy (XAS) (BL. 2.2, SLRI, Thailand), respectively. The morphological and microstructural characteristics of the prepared materials were studied using transmission electron microscopy (TEM) (FEI, TECNAI G2 20) and scanning electron microscopy (FEI, Helios Nano Lab G3 C). To estimate the domain sizes of Li_2_MnO_3_ and LiCoO_2_, HRTEM images showing clear Li_2_MnO_3_ and LiCoO_2_ domains were selected. Then, the selected domains were carefully traced. Afterwards, the areas inside the traced figures were determined using the measuring function of ImageJ software. The 2D areas, obtained from several Li_2_MnO_3_ and LiCoO_2_ domains of at least five individual particles with sizes of around 100 nm were averaged. Moreover, X'Pert HighScore Plus software was used to identify the phases of the synthesized materials from the obtained x-ray diffraction patterns. Rietveld refinement was done to calculate lattice parameters. Mn–O, Mn-TM, Co–O, and Co-TM bond lengths were obtained by EXAFS fitting using Artemis software.

### Electrochemical characterization

Electrodes were fabricated using a mixture of the synthesized cathode materials, super P carbon black (Alfa Aesar) as a conductive agent, and polyvinylidene fluoride (PVDF, Arkema) as a binder. Synthesized cathode materials, super P carbon black, and polyvinylidene fluoride (PVDF, Arkema) were dissolved in a N-methyl-2-pyrollidone (NMP, Aldrich) solvent at a mass ratio of 80:10:10. Required amounts of these materials were mixed in a shaker for 2 h to obtain a slurry. This slurry was coated onto an aluminum foil using a doctor blade after which it was further dried in a vacuum oven at 80 °C. Haft cells were assembled in an Argon-filled glove box using Swagelok cases. Metallic lithium foil (Alfa Aesar) and 1 M LiPF6 in a 4:3:3 volume ratio of ethylene carbonate (EC): dimethyl carbonate (DMC): diethyl carbonate (DEC) (MTI) were used as the negative electrode and electrolyte, respectively. The separator was made of Celgard 2400. Electrochemical performance including specific capacity, cycling stability, and rate capability of the prepared cathodes was investigated in galvanostatic cycling tests (NEWARE, BTS-4008). Moreover, electrochemical impedance spectroscopy (EIS) (Corrtest, CS310) and a galvanostatic intermittent titration technique (GITT) (NEWARE, BTS-4008) were used to study the electronic conductivity and lithium-ion diffusion coefficients of the prepared electrode materials, respectively.

## Supplementary Information

Supplementary Information.

## Data Availability

The datasets used and/or analysed during the current study available from the corresponding author on reasonable request.
