# Supplementary material for: Impacts of Mg doping on the structural properties and degradation mechanisms of a Li and Mn rich layered oxide cathode for lithium-ion batteries
Source: Sci Rep. 2023 Mar 20;13:4526. doi: 10.1038/s41598-023-31492-0 (PMC10027840; doi:10.1038/s41598-023-31492-0)
Supplement: Supplementary file 1 — Supplementary Information. [file 41598_2023_31492_MOESM1_ESM.pdf]

Supplementary information

Impacts of Mg doping on the Structural Properties and Degradation Mechanisms of a Li and Mn Rich Layered Oxide Cathode Material for Lithium-ion Batteries

Songyoot Kaewmala[a], Natthapong Kamma[2], Sunisa Buakeaw[3], Wanwisa Limphirat[4], Jeffrey Nash[1], Sutham Srilomsak[1, 2], Pimpa Limthongkul[3], and Nonglak Meethong[1, 2, *]

[1]Institute of Nanomaterials Research and Innovation for Energy (IN-RIE), Khon Kaen University, Khon Kaen 40002, Thailand

[2]Materials Science and Nanotechnology Program, Department of Physics, Faculty of Science, Khon Kaen University, Khon Kaen 40002, Thailand

[3]National Energy Technology Center, National Science and Technology Development Agency, 111 Thailand Science Park, Phaholyothin Rd., Klong 1, Klong Luang, Pathumthani, 12120, Thailand

[4]Synchrotron Light Research Institute, 111 University Avenue, Suranaree, Muang, Nakhon Ratchasima 30000, Thailand

* nonmee@kku.ac.th (N.M.)

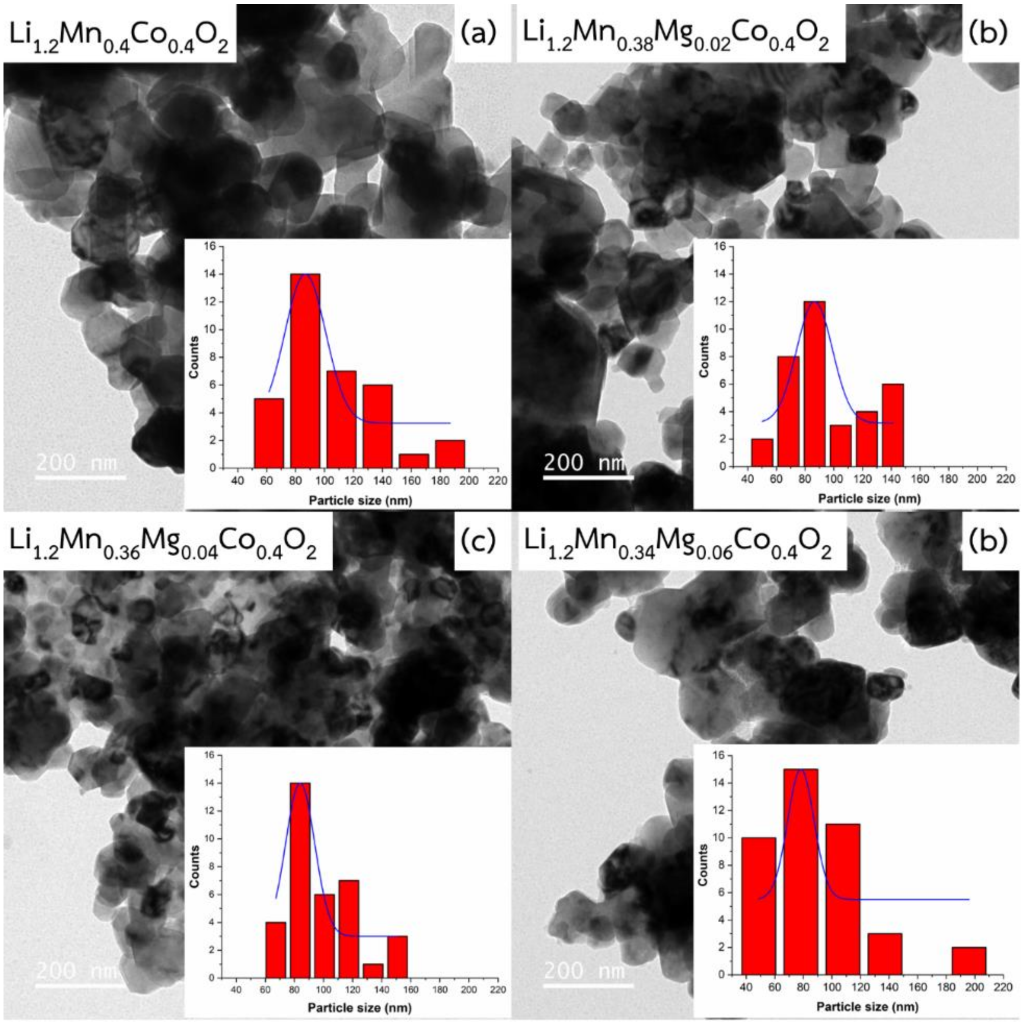

Figure S1. TEM images of the $Li_{1.2}Mn_{0.4-x}$ $Mg_xCo_{0.4}O_2$ (x=0.00, 0.02, 0.04, and 0.06) materials.

Table S1. The averages Li_2MnO-like and $LiCoO_2$-like domain sizes of pristine $Li_{1.2}Mn_{0.4}Co_{0.4}O_2$ and Mg-doped $Li_{1.2}Mn_{0.4}Co_{0.4}O_2$ materials, obtained from at least five individual particles with particle sizes of around 100 nm which is close to the average particle size of the cathode materials to make a reasonable comparison.

Sample	Average Li_2MnO_3-like domain size (nm^2)	Average $LiCoO_2$-like domain size (nm^2)
$Li_{1.2}Mn_{0.4}Co_{0.4}O_2$	>1000	>1300
$Li_{1.2}Mn_{0.38}Mg_{0.02}Co_{0.4}O_2$	600	600
$Li_{1.2}Mn_{0.36}Mg_{0.04}Co_{0.4}O_2$	400	500
$Li_{1.2}Mn_{0.34}Mg_{0.06}Co_{0.4}O_2$	200	160

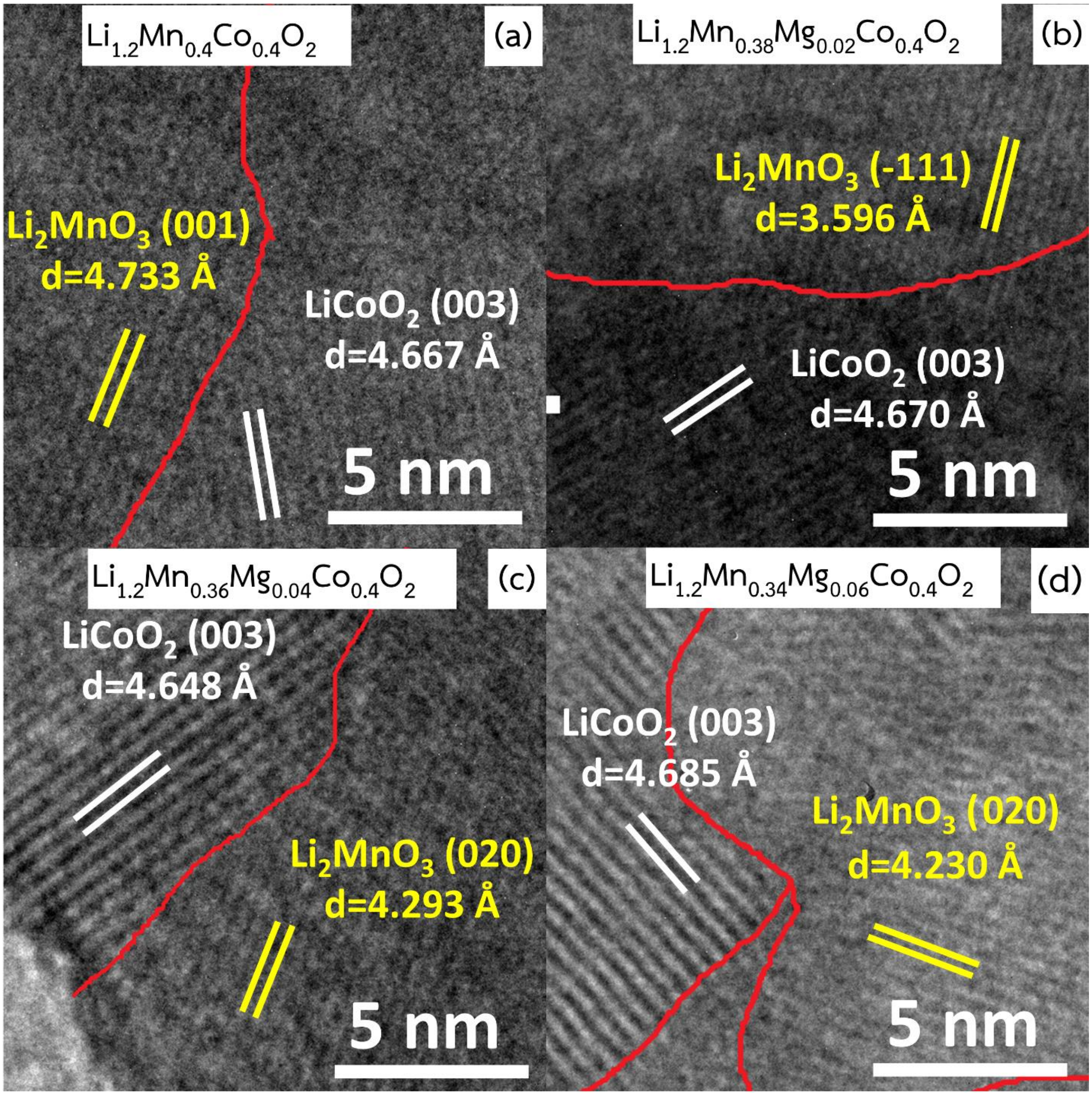

Figure S2. Images show the presence of Li_2MnO_3 and $LiCoO_2$ regions in individual particles of the prepared cathodes, corresponding to the dash green rectangles as demonstrated in Fig. 1.

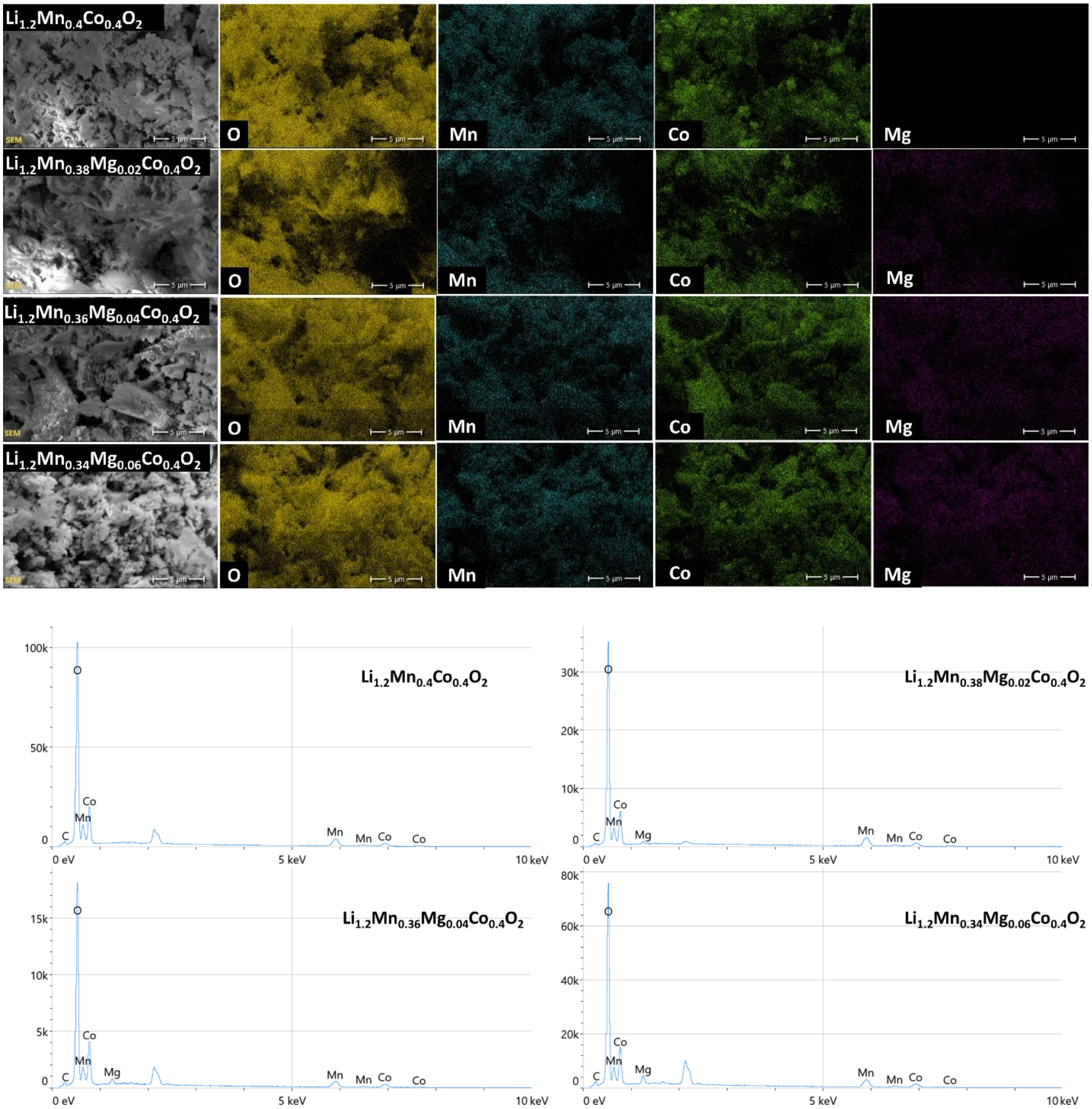

Figure S3. Elemental mapping and EDS spectra of synthesized materials obtained from an EDS technique.

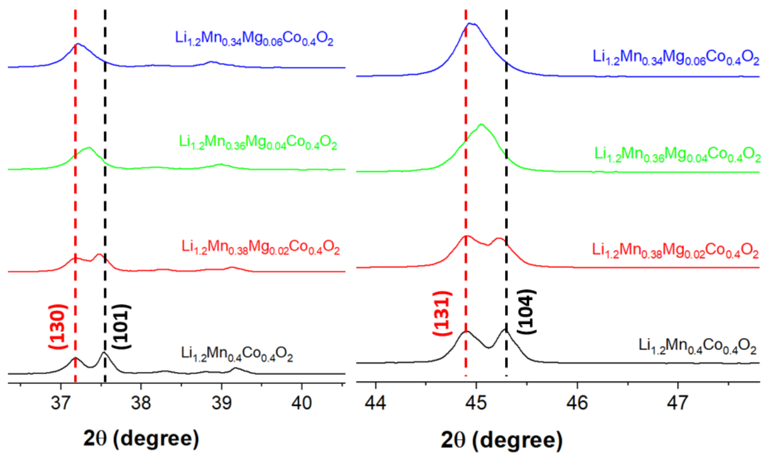

Figure S4. Comparison of X-ray diffraction spectra of the $Li_{1.2}Mn_{0.4-x}Mg_xCo_{0.4}O_2$ (x=0.00, 0.02, 0.04, and 0.06) materials at 2θ values of approximately 37.5° and 45.0°.

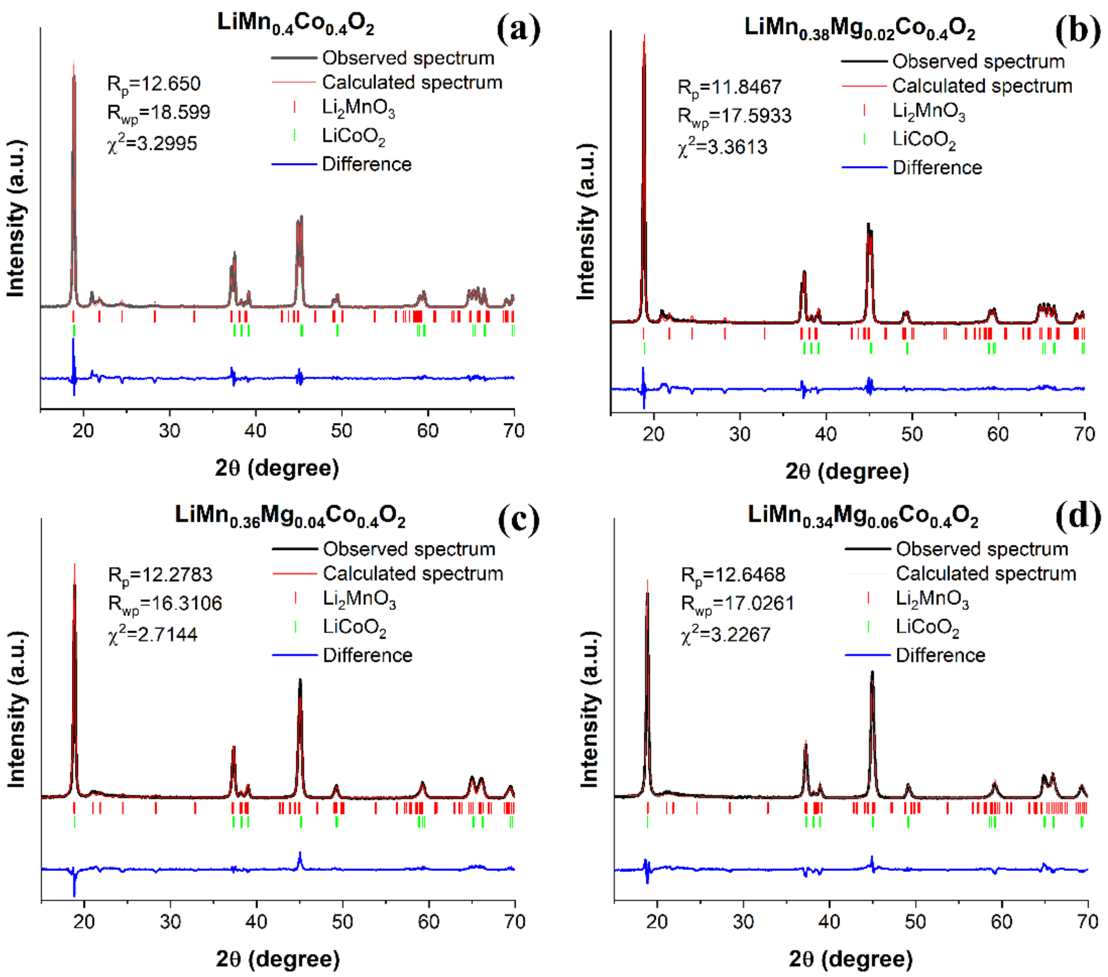

Figure S5. Rietveld refinement results of pristine $Li_{1.2}Mn_{0.4}Co_{0.4}O_2$ (a), $Li_{1.2}Mn_{0.38}Mg_{0.02}Co_{0.4}O_2$ (b), $Li_{1.2}Mn_{0.36}Mg_{0.04}Co_{0.4}O_2$ (c), and $Li_{1.2}Mn_{0.34}Mg_{0.06}Co_{0.4}O_2$ (d) materials

Table. S2 Atomic positions obtained from Rietveld refinement of pristine $Li_{1.2}Mn_{0.4}Co_{0.4}O_2$ and Mg-dopped $Li_{1.2}Mn_{0.4}Co_{0.4}O_2$ materials.

Sample	Li_2MnO_3						$LiCoO_2$					
	Element	Wyckoff position	X	Y	Z	Occupancy	Element	Wyckoff position	X	Y	Z	Occupancy
$Li_{1.2}Mn_{0.4}Co_{0.4}O_2$	Li1	2a	0.0000	0.0000	0.0000	0.7300	Co	3b	0.0000	0.0000	0.5000	1.0000
	Mn1	2a	0.0000	0.0000	0.0000	0.2700	Li	3a	0.0000	0.0000	0.0000	1.0000
	Li2	2d	0.0000	0.5000	0.5000	1.0000	O	6c	0.0000	0.0000	0.2395	1.0000
	Li3	4h	0.0000	0.0159	0.5000	1.0000						
	Mn2	4g	0.0000	0.3308	0.0000	0.8700						
	Li4	4g	0.0000	0.3308	0.0000	0.1300						
	O1	4i	0.7197	0.0000	0.2251	1.0000						
	O2	8i	0.2524	0.1765	0.2234	1.0000						
$Li_{1.2}Mn_{0.38}Mg_{0.02}Co_{0.4}O_2$	Li1	2a	0.0000	0.0000	0.0000	0.7300	Co	3b	0.0000	0.0000	0.5000	1.0000
	Mn1	2a	0.0000	0.0000	0.0000	0.2700	Li	3a	0.0000	0.0000	0.0000	1.0000
	Li2	2d	0.0000	0.5000	0.5000	1.0000	O	6c	0.0000	0.0000	0.2395	1.0000
	Li3	4h	0.0000	0.0159	0.5000	1.0000						
	Mn2	4g	0.0000	0.3308	0.0000	0.8300						
	Li4	4g	0.0000	0.3308	0.0000	0.1300						
	O1	4i	0.7197	0.0000	0.2251	1.0000						
	O2	8i	0.2524	0.1765	0.2234	1.0000						
	Mg	4g	0.0000	0.3308	0.0000	0.0200						
$Li_{1.2}Mn_{0.36}Mg_{0.04}Co_{0.4}O_2$	Li1	2a	0.0000	0.0000	0.0000	0.7300	Co	3b	0.0000	0.0000	0.5000	1.0000
	Mn1	2a	0.0000	0.0000	0.0000	0.2700	Li	3a	0.0000	0.0000	0.0000	1.0000
	Li2	2d	0.0000	0.5000	0.5000	1.0000	O	6c	0.0000	0.0000	0.2395	1.0000
	Li3	4h	0.0000	0.0159	0.5000	1.0000						
	Mn2	4g	0.0000	0.3308	0.0000	0.7900						
	Li4	4g	0.0000	0.3308	0.0000	0.1300						
	O1	4i	0.7197	0.0000	0.2251	1.0000						
	O2	8i	0.2524	0.1765	0.2234	1.0000						
	Mg	4g	0.0000	0.3308	0.0000	0.0800						
$Li_{1.2}Mn_{0.34}Mg_{0.06}Co_{0.4}O_2$	Li1	2a	0.0000	0.0000	0.0000	0.7300	Co	3b	0.0000	0.0000	0.5000	1.0000
	Mn1	2a	0.0000	0.0000	0.0000	0.2700	Li	3a	0.0000	0.0000	0.0000	1.0000
	Li2	2d	0.0000	0.5000	0.5000	1.0000	O	6c	0.0000	0.0000	0.2395	1.0000
	Li3	4h	0.0000	0.0159	0.5000	1.0000						
	Mn2	4g	0.0000	0.3308	0.0000	0.7500						
	Li4	4g	0.0000	0.3308	0.0000	0.1300						
	O1	4i	0.7197	0.0000	0.2251	1.0000						
	O2	8i	0.2524	0.1765	0.2234	1.0000						
	Mg	4g	0.0000	0.3308	0.0000	0.1200						

Table. S3 Comparisons of the calculated lattice constants of pristine $Li_{1.2}Mn_{0.4}Co_{0.4}O_2$ and Mg-doped $Li_{1.2}Mn_{0.4}Co_{0.4}O_2$ materials

Conditions	Lattice parameters							
	Li_2MnO_3 (C2/m) $\alpha = \gamma = 90°$					$LiCoO_2$ ($R\bar{3}m$) $\alpha = \beta = 90°$ and $\gamma = 120°$		
	a (Å)	b (Å)	c (Å)	β (°)	Unit cell volume (Å^3)	a and b (Å)	C (Å)	Unit cell volume (Å^3)
$Li_{1.2}Mn_{0.4}Co_{0.4}O_2$	4.9245 ±0.0007	8.5126 ±0.0011	5.0088 ±0.0006	108.9836±0.0114	198.5489	2.8130±0.0001	14.1361±0.0013	96.8998
$Li_{1.2}Mn_{0.38}Mg_{0.02}Co_{0.4}O_2$	4.9210 ±0.0007	8.5028 ±0.0013	5.0061 ±0.0007	108.9851±0.0133	198.0718	2.8149±0.0001	14.1290±0.0017	96.9527
$Li_{1.2}Mn_{0.36}Mg_{0.04}Co_{0.4}O_2$	4.9223 ±0.0009	8.4834 ±0.0023	5.0026 ±0.0010	108.9781±0.0203	197.5434	2.8138±0.0001	14.1434±0.0006	97.7526
$Li_{1.2}Mn_{0.34}Mg_{0.06}Co_{0.4}O_2$	4.9262 ±0.0009	8.4594 ±0.0013	4.9766 ±0.0008	108.5603±0.0136	196.6003	2.8361±0.0002	14.1883±0.0008	98.8321

Table S4. The bond distances and agreement indices obtained from XAS spectra fitting of EXAFS spectra of pristine $Li_{1.2}Mn_{0.4}Co_{0.4}O_2$ and Mg-doped $Li_{1.2}Mn_{0.4}Co_{0.4}O_2$ materials

Li_2MnO_3 structure (Mn *K*-edge)						
Sample	Shell	N (atoms)	R (Å)	σ^2 (Å^2)	R-factor	E_0 (eV)
$LiMn_{0.4}Co_{0.4}O_2$	O	6	1.961±0.010	0.001 (set)	0.004	5.817
	Mn	3	2.966±0.015	0.003±0.002		
$LiMn_{0.38}Mg_{0.02}Co_{0.4}O_2$	O	6	1.949±0.010	0.001(set)	0.004	5.817
	Mn	3	2.964±0.017	0.004±0.003		
$LiMn_{0.36}Mg_{0.04}Co_{0.4}O_2$	O	6	1.945±0.013	0.001(set)	0.003	5.621
	Mn	3	2.961±0.022	0.005±0.003		
$LiMn_{0.34}Mg_{0.06}Co_{0.4}O_2$	O	6	1.936±0.013	0.001(set)	0.012	5.803
	Mn	3	2.952±0.020	0.003±0.003		
$LiCoO_2$ structure (Co *K*-edge)						
Sample	Shell	N (atoms)	R (Å)	σ^2 (Å^2)	R-factor	E_0 (eV)
$LiMn_{0.4}Co_{0.4}O_2$	O	6	1.870±0.013	0.012±0.008	0.010	9.500 (set)
	Co	6	2.791±0.020	0.004±0.006		
$LiMn_{0.38}Mg_{0.02}Co_{0.4}O_2$	O	6	1.878±0.013	0.008±0.009	0.012	9.500 (set)
	Co	6	2.789±0.020	0.004±0.007		
$LiMn_{0.36}Mg_{0.04}Co_{0.4}O_2$	O	6	1.879±0.013	0.009±0.007	0.007	9.500 (set)
	Co	6	2.799±0.020	0.006±0.006		
$LiMn_{0.34}Mg_{0.06}Co_{0.4}O_2$	O	6	1.880±0.013	0.011±0.008	0.008	9.500 (set)
	Co	6	2.794±0.020	0.006±0.006		

Table. S5 Comparison of calculated resistances obtained from fitting parameters using the equivalent circuit model illustrated in Fig. S7 of the fresh cell and the cells cycled at 0.3C after cycling for 100 cycles

Sample	Fresh cell		After 100 cycles	
	R_Ω (Ω)	R_{CT} (Ω)	R_Ω (Ω)	R_{CT} (Ω)
$LiMn_{0.4}Co_{0.4}O_2$	0.432	9.235	0.575	739.485
$LiMn_{0.38}Mg_{0.02}Co_{0.4}O_2$	0.410	8.452	0.521	345.214
$LiMn_{0.36}Mg_{0.04}Co_{0.4}O_2$	0.472	8.324	0.554	305.236
$LiMn_{0.34}Mg_{0.06}Co_{0.4}O_2$	0.484	8.624	0.601	479.258

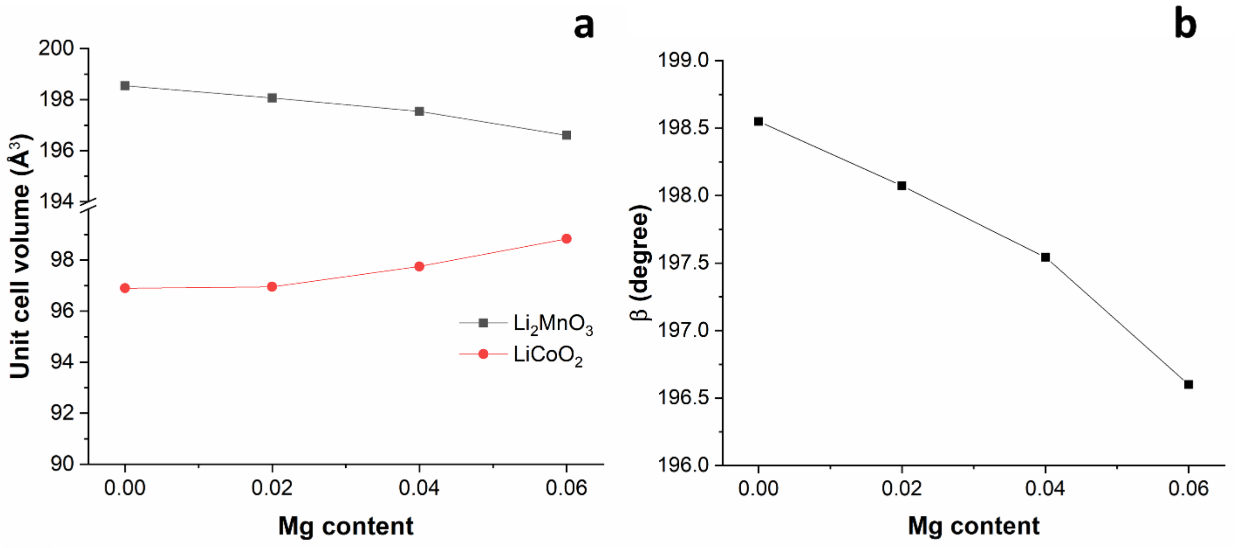

Figure S6. Calculated unit cell volume (a) and β (b) of pristine $Li_{1.2}Mn_{0.4}Co_{0.4}O_2$ and Mg-doped $Li_{1.2}Mn_{0.4}Co_{0.4}O_2$ materials.

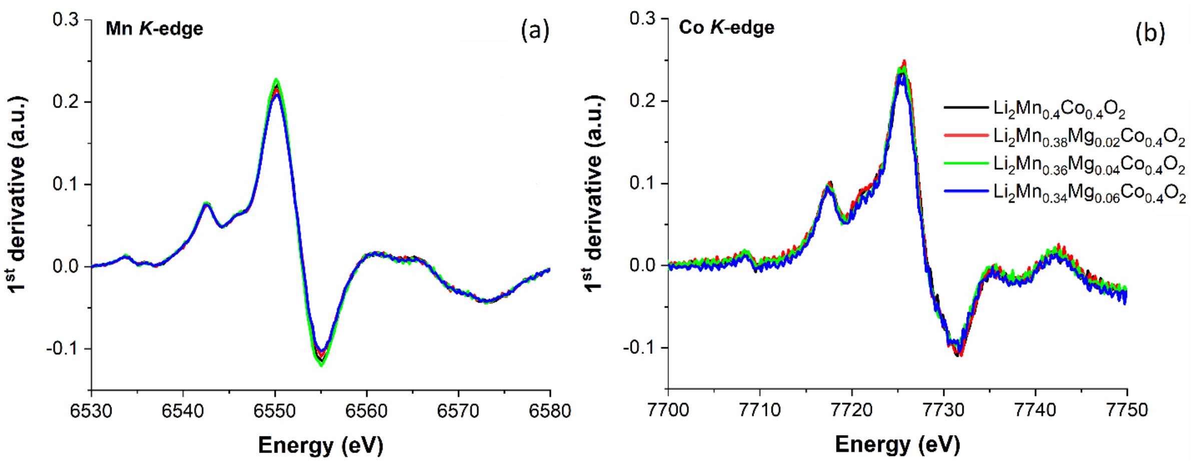

Figure S7. 1st derivative spectra at the Mn (a) and Co (b) *K*-edges of pristine $Li_{1.2}Mn_{0.4}Co_{0.4}O_2$, Mg-doped $Li_{1.2}Mn_{0.4}Co_{0.4}O_2$, Li_2MnCoO_3, and $LiCoO_2$ materials

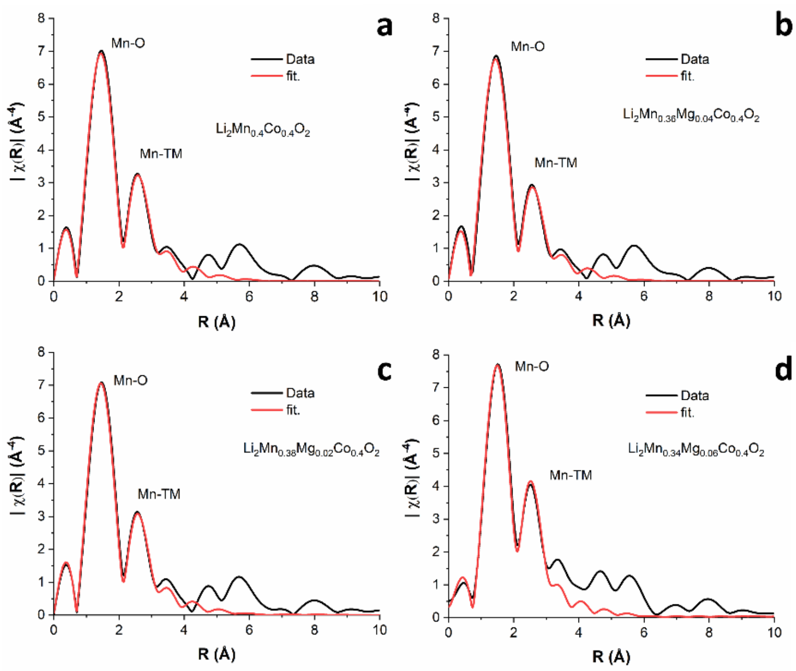

Figure S8. Mn *K*-edges EXAFS fitting results of pristine $Li_{1.2}Mn_{0.4}Co_{0.4}O_2$ (a), $Li_{1.2}Mn_{0.38}Mg_{0.02}Co_{0.4}O_2$ (b), $Li_{1.2}Mn_{0.36}Mg_{0.04}Co_{0.4}O_2$ (c), and $Li_{1.2}Mn_{0.34}Mg_{0.06}Co_{0.4}O_2$ (d) materials

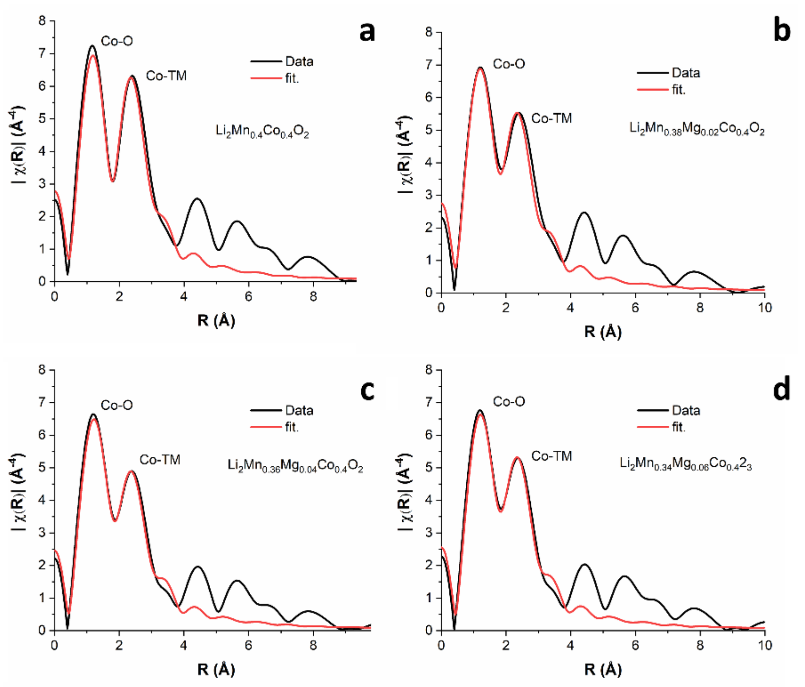

Figure S9. Co *K*-edges EXAFS fitting results of pristine $Li_{1.2}Mn_{0.4}Co_{0.4}O_2$ (a), $Li_{1.2}Mn_{0.38}Mg_{0.02}Co_{0.4}O_2$ (b), $Li_{1.2}Mn_{0.36}Mg_{0.04}Co_{0.4}O_2$ (c), and $Li_{1.2}Mn_{0.34}Mg_{0.06}Co_{0.4}O_2$ (d) materials

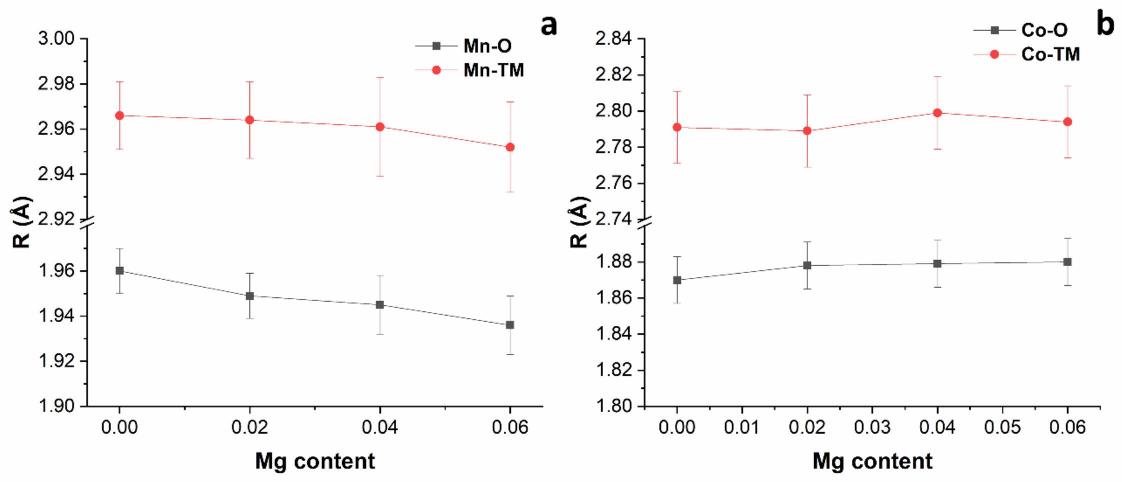

Figure S10. Calculated Mn-O, Mn-TM, Co-O, and Co-TM bond lengths of pristine $Li_{1.2}Mn_{0.4}Co_{0.4}O_2$ and Mg-doped $Li_{1.2}Mn_{0.4}Co_{0.4}O_2$ materials.

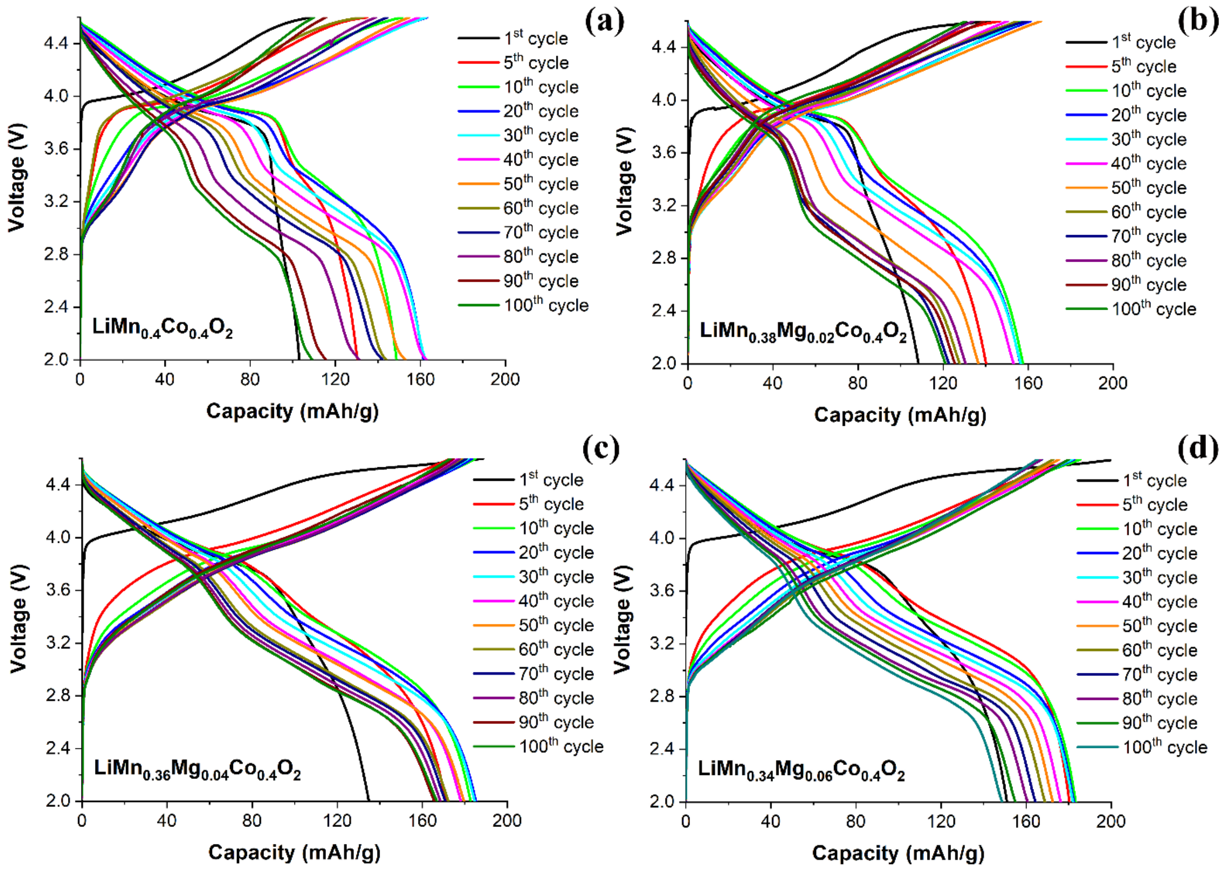

Figure S11. Voltage profiles of pristine $Li_{1.2}Mn_{0.4}Co_{0.4}O_2$ and Mg-doped $Li_{1.2}Mn_{0.4}Co_{0.4}O_2$ materials cycled at C/3.

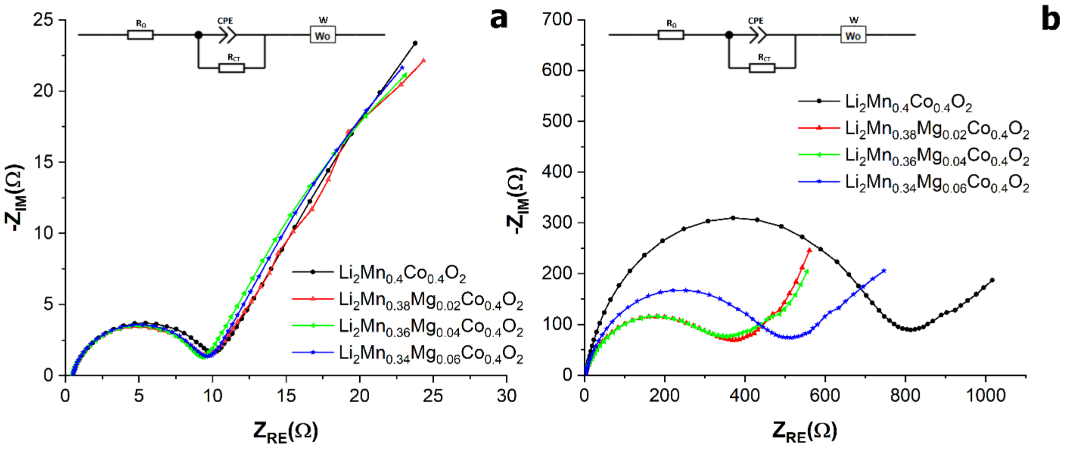

Figure S12. Nyquist plots of the fresh cell and the cells cycled at 0.3C after cycling for 100 cycles and the inset shows an equivalent circuit. R_{Ω}, R_{CT}, CPE, and W denote the ohmic resistance, the charge transfer resistance, constant phase element, and Warburg resistance, respectively.

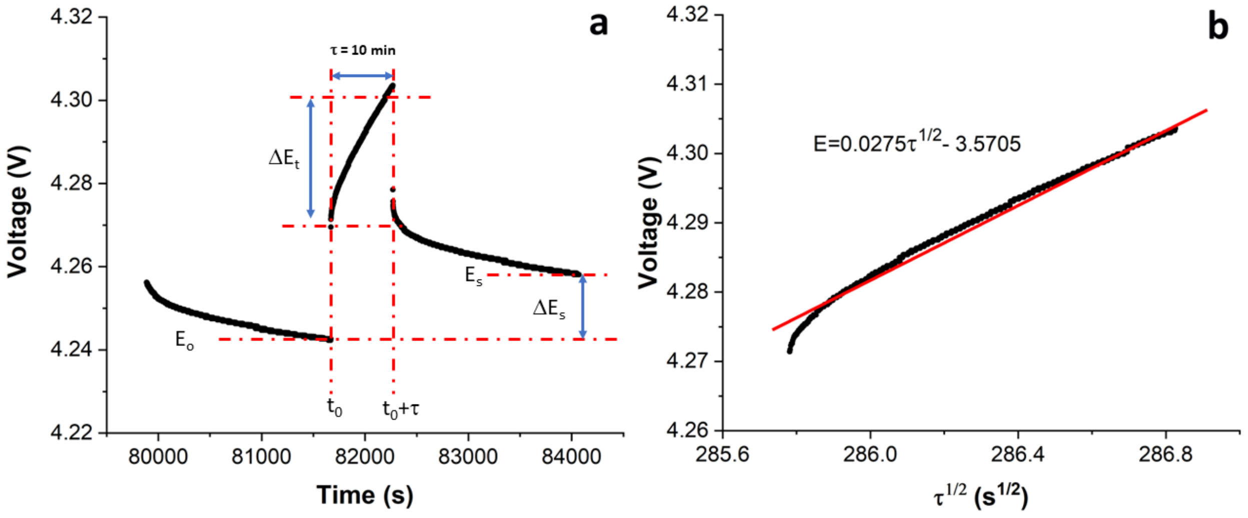

Figure S13. Single titration at about 4.26 V during GITT measurement (a) and the cell voltage as a function of $t^{1/2}$ (b)

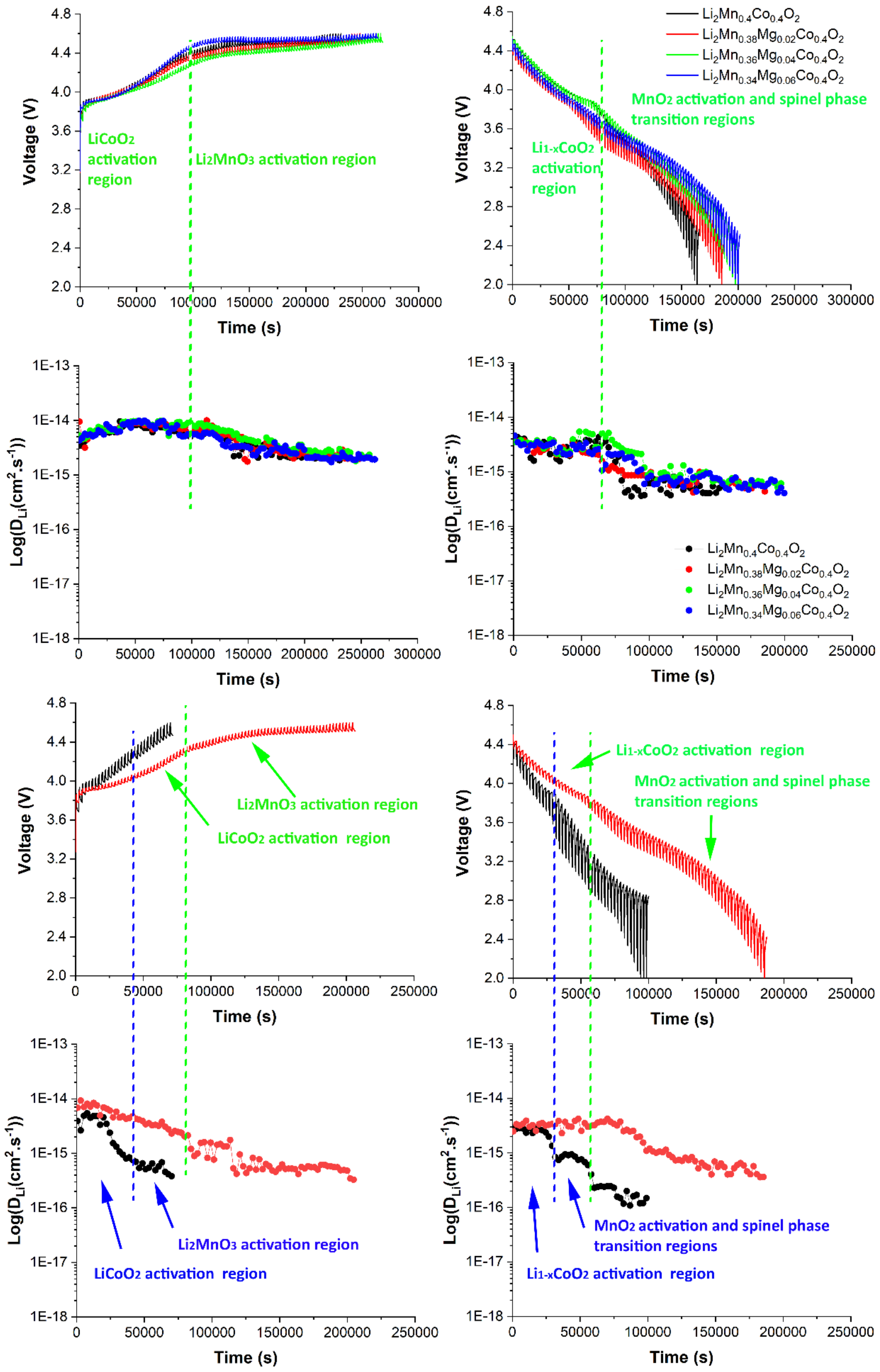

Figure S14. GITT profiles and calculated lithium-ion diffusion coefficients as a function of time of a pristine $Li_{1.2}Mn_{0.4}Co_{0.4}O_2$ and Mg-doped $Li_{1.2}Mn_{0.4}Co_{0.4}O_2$ materials at the 1st and 100th cycles for charging (a and c) and discharging (b and d) processes
